# Elusive search for effective provider interventions: a systematic review of provider interventions to increase adherence to evidence-based treatment for depression

**DOI:** 10.1186/s13012-018-0788-8

**Published:** 2018-07-20

**Authors:** Eric R. Pedersen, Lisa Rubenstein, Ryan Kandrack, Marjorie Danz, Bradley Belsher, Aneesa Motala, Marika Booth, Jody Larkin, Susanne Hempel

**Affiliations:** 1 RAND Corporation, 1776 Main Street, PO Box 2138, Santa Monica, CA 90407 USA; 2 RAND Corporation, Pittsburgh, USA; 3 David Geffen School of Medicine at UCLA, Los Angeles, USA; 4 UCLA Fielding School of Public Health, Los Angeles, USA; 5 Psychological Health Center of Excellence, Defense Health Agency, Falls Church, USA; 6 Uniformed Services University of the Health Sciences, Department of Psychiatry, Bethesda, USA

**Keywords:** Depression, Provider intervention, Guidelines, Evidence-based, Major depressive disorder, Primary care, Specialty care

## Abstract

**Background:**

Depression is a common mental health disorder for which clinical practice guidelines have been developed. Prior systematic reviews have identified complex organizational interventions, such as collaborative care, as effective for guideline implementation; yet, many healthcare delivery organizations are interested in less resource-intensive methods to increase provider adherence to guidelines and guideline-concordant practices. The objective of this systematic review was to assess the effectiveness of healthcare provider interventions that aim to increase adherence to evidence-based treatment of depression in routine clinical practice.

**Methods:**

We searched five databases through August 2017 using a comprehensive search strategy to identify English-language randomized controlled trials (RCTs) in the quality improvement, implementation science, and behavior change literature that evaluated outpatient provider interventions, in the absence of practice redesign efforts, to increase adherence to treatment guidelines or guideline-concordant practices for depression. We used meta-analysis to summarize odds ratios, standardized mean differences, and incidence rate ratios, and assessed quality of evidence (QoE) using the GRADE approach.

**Results:**

Twenty-two RCTs promoting adherence to clinical practice guidelines or guideline-concordant practices met inclusion criteria. Studies evaluated diverse provider interventions, including distributing guidelines to providers, education/training such as academic detailing, and combinations of education with other components such as targeting implementation barriers. Results were heterogeneous and analyses comparing provider interventions with usual clinical practice did not indicate a statistically significant difference in guideline adherence across studies. There was some evidence that provider interventions improved individual outcomes such as medication prescribing and indirect comparisons indicated more complex provider interventions may be associated with more favorable outcomes. We did not identify types of provider interventions that were consistently associated with improvements across indicators of adherence and across studies. Effects on patients’ health in these RCTs were inconsistent across studies and outcomes.

**Conclusions:**

Existing RCTs describe a range of provider interventions to increase adherence to depression guidelines. Low QoE and lack of replication of specific intervention strategies across studies limited conclusions that can be drawn from the existing research. Continued efforts are needed to identify successful strategies to maximize the impact of provider interventions on increasing adherence to evidence-based treatment for depression.

**Trial registration:**

PROSPERO record CRD42017060460 on 3/29/17

**Electronic supplementary material:**

The online version of this article (10.1186/s13012-018-0788-8) contains supplementary material, which is available to authorized users.

## Background

Depression is one of the most common mental health disorders worldwide, affecting about 7% of the adult populations in the USA and the European Union [1, 2]. Depression is associated with poor quality of life and significantly decreased psychosocial functioning [3]; high societal costs related to patient care, unstable or unproductive employment, marital and relationship disruption [4–6]; and mortality [7, 8]. Depression is most often identified by practitioners in primary care settings [9, 10]. Collaborative care interventions in primary care can significantly and cost-effectively improve depression care outcomes [11–14] and can improve adherence to clinical guidelines for effective psychological and pharmacological treatments for depression [15–17]. However, collaborative care interventions require major commitment to organizational change, including commitment by mental health specialists to support the revamped system. Levels of organizational [18] and provider [19] readiness significantly influence any potential positive effect of collaborative care on outcomes. Given that not all organizations will have the resources or readiness to implement large system redesign efforts, it is important to understand how and whether less intensive intervention efforts that may be easier to adopt can influence provider behavior.

Although most complex interventions that aim to improve depression care include some elements related to guideline-based education [20–24], further research is needed to evaluate the comparative effects of different educational interventions, which do not require organization change, on specific provider behaviors. Knowledge transfer is a burgeoning field that seeks to reduce the gap between research on evidence-based interventions and use of these interventions by generating, sharing, and applying research knowledge in practice [25]. However, knowledge transfer work is only beginning to systematically address methods for achieving clinical guideline-based provider behavior change. Based on conclusions that passive dissemination in educational and quality assurance interventions (e.g., mailing guidelines to providers with no reminders or follow-up) is generally ineffective [20], researchers have emphasized system-level strategies that require restructuring care processes, extensive time for planning, financial reorganization, and establishing new clinics and staff [26, 27]. Yet, education and dissemination interventions may have greater feasibility than large-scale organizational change, may be necessary for promoting organizational and provider readiness, and are often critical components of the broader system-level interventions. In addition, in settings outside of primary care, there is still much reliance on direct knowledge transfer paradigms. Adoption of evidence-based care and fidelity to manualized treatment are among the biggest challenges in specialty care settings [28, 29].

Two prior reviews of organizational and education interventions implemented exclusively in primary care settings concluded that provider training alone does not improve depression care [30, 31]. The more comprehensive review [30] was conducted in 2003, and updated reviews are necessary to increase understanding of what effective behavior change strategies [20] can promote adherence to guidelines and guideline-concordant practices in specific settings. The most recent review [31] focused only on physician (e.g., general practitioners) education and did not address other interventions or providers, including those working in specialty care settings. Moreover, neither review focused on guideline adherence by providers; instead, they included only patient outcomes. Improved guideline adherence is a critical step in the path toward depression outcome improvement. Thus, the results of interventions targeting provider behavior change are important to policy makers, administrators, and providers in assessing how best to increase the use of evidence-based care for depression. Lastly, both reviews assessed only interventions within primary care settings: Since depression is most often identified by practitioners in primary care settings [9, 10], understanding which provider interventions work in these settings is essential. However, the majority of RCTs evaluating medication and behavioral treatments for depression are conducted in specialty care settings [32–35]. Therefore, specialty care settings (e.g., managed behavioral health care organizations, psychiatry private practice) are also important settings in which to assess the effectiveness of interventions [36].

In this systematic review, we synthesize estimates of the effects of provider interventions, with a specific focus on behavioral health provider change, to promote adherence to evidence-based treatments for depression. We purposefully focus on RCTs with provider outcomes as the primary outcome, and we include both specialty and primary care settings, given the large number of behavioral change strategies that have been proposed to encourage providers to adopt evidence-based treatments for depression in practice [30, 37–39]. We also examine whether provider intervention effects vary across provider target of the intervention (i.e., a sole provider or a team of providers).

## Methods

### Registration

The review is based on a registered systematic review protocol (PROSPERO record CRD42017060460).

### Search strategy

In August 2017, we searched the databases PubMed, PsycINFO, the Cumulative Index of Nursing and Allied Health Literature, the Cochrane Central Register of Controlled Trials, and the Cochrane Database of Systematic Reviews to identify English-language reports of RCTs that evaluated the effects of provider interventions. Searches included depression terms (e.g., depress$, mood dysregulation), general terms for knowledge transfer and organizational quality improvement (e.g., evidence-based guideline, research to practice) [40], terms related to provider interventions for clinical practice guidelines and implementation strategies (e.g., academic detailing, reminder systems), approaches for continuous quality improvement (e.g., CQI; quality manage$, model for improvement), terms for continuous professional education (e.g., continuing education, learning collaborative), and behavior change terms (e.g., reframing, incentive) [24, 41–43] (see Additional file 1: Appendix A for full search strategy). We also searched bibliographies of existing systematic reviews and included studies.

### Eligibility

Eligible *participants* were healthcare providers responsible for patient care in the outpatient setting (e.g., primary care physicians, psychiatrists, mental health professionals, nurse practitioners, other general practitioners such as physician assistants). Eligible *interventions* aimed to increase adherence to depression guidelines and guideline-concordant practice (e.g., continuing education, quality improvement projects, and financial, organizational, or regulatory interventions that used knowledge translation strategies). To determine the effect of interventions on provider behavior change, we excluded studies that primarily assessed the effects of large system redesign efforts, such as collaborative care, where new clinics are established, care is reorganized (e.g., implementing dedicated care managers), and training of existing providers is only a minor component of the larger intervention. We also included interventions aimed at improving depression treatment and excluded studies focused solely on improving screening/assessment or referral behavior. Eligible *comparators* were no intervention, usual care practice (UCP), wait list control, or other provider interventions (e.g., organizational system redesign or an out of scope intervention). *Outcomes* documented the adherence of providers to guidelines or to guideline-concordant practices. We evaluated observable, objective changes in provider behavior because they are better markers of intervention success than are provider knowledge, attitudes, satisfaction, or perceived changes, which occur earlier in the change process [44], and while they are often precursors to change, they may not progress to the observable changes in behavior necessary for impacting patient outcomes. *Timing* involved any intervention duration and any follow-up period, and *setting* was any outpatient healthcare delivery facility or other physician practice setting. The review was restricted to RCT *study design*, with studies randomizing provider participants or practice sites to interventions. We aimed to identify the presence and absence of evidence from this robust research design, which allows for the development of the confident evidence statements desired for policy changes.

### Data extraction and critical appraisal

We used a standardized approach for systematic reviews with detailed instructions for reviewers to reduce ambiguities. Following a pilot session to ensure similar interpretation of the inclusion and exclusion criteria, two reviewers independently screened all titles and abstracts of retrieved citations. Citations judged as potentially eligible by one or both reviewers were obtained as full text. The reviewers then both screened full-text publications against the specified inclusion and exclusion criteria, abstracted data from those studies that met the inclusion criteria, and assessed their risk of bias. All disagreements were resolved through author discussions. Critical appraisal assessments included the Cochrane Risk of Bias tool [45] and the Quality Improvement Minimum Quality Criteria Set (QI-MQCS) [46] to address internal validity and study-design independent criteria for interventions aiming to improve healthcare.

### Analytic plan

We used the Hartung-Knapp-Sidik-Jonkman method for random effects meta-analysis to summarize odds ratios (OR), standardized mean differences (SMD), and incidence rate ratios (IRR) together with the 95% confidence interval (CI). Tests of heterogeneity were performed using the *I*^2^ statistic [47]. Values of *I*^2^ of 30–60% possibly represent moderate heterogeneity, 50–90% substantial heterogeneity, and 75–100% considerable heterogeneity [48]. We assessed the quality of evidence (QoE) using the Grades of Recommendation, Assessment, Development, and Evaluation (GRADE) approach.

The identified studies assessed a variety of study-specific outcomes. To facilitate comparisons across studies with the available data, we selected a dichotomous, continuous, and incidence rate variable as the main indications of adherence to depression guidelines or guideline-concordant practices (see Table 1). The lead author reviewed the intervention content and selected these main indicators of adherence from a list of all reported outcomes in the study. A content expert checked the selection. To avoid bias, the specific outcomes were selected before analyses. We also analyzed individual provider behavior change outcomes that were reported in more than one study: medication prescribing, contact with patients, specific intervention adherence, and referral to mental healthcare specialists offered to patients. We analyzed the effects on patient outcomes when available. We differentiated comparators as UCP (e.g., no intervention or interventions not aimed at depression treatment), practice redesign efforts (e.g., introduction of a nurse disease manager or a continuous quality improvement team), or other active provider interventions (e.g., training providers in a specific type of behavioral therapy, such as motivational interviewing [49]).

**Table 1:** Evidence table of included studies

Study details	Participants	Intervention and treatment	Outcomes and results
Aakhus [84]; Aakhus, 2014 [85] Country: Norway Setting: primary care office Randomization unit: municipality	Number of sites: 51 municipalities (26 intervention, 28 control) Providers: 124 (51 intervention, 73 control) PCPs Patients: 134 (66 intervention, 68 control) home-dwelling elderly patients 65 years or older Diagnosis: depression (clinical diagnosis)	Intervention: outreach visits to GPs; website that provided recommendations, tools to diagnose and manage elderly patients with depression, and online courses; CME course approved by the Norwegian Medical Association; tailored information based on profession or relation to the healthcare Comparator: UC Timing of follow-up: 8 months	Provider behaviors: Mean adherence to recommendations for the management of depression at 8 months* MD − 5.00 (95% CI − 11.87, 1.87) Patient health outcomes: Adherence to antidepressant > 0 at 8 months OR 1.33 (95% CI 0.88, 2.00) GP assessed CGI-I at 8 months MD 0.03 (95% CI − 0.20, 0.26) HADS depression at 8 months MD − 0.28 (95% CI − 1.87, 1.31) Patient assessed PGI at 8 months MD 0.10 (95% CI − 0.38, 0.58)
Azocar [86] Country: USA Setting: managed behavioral health care organization (specialty care) Randomization unit: provider	Number of sites: NR Providers: 443 (162 guidelines only, 132 targeted guidelines, 149 control) mental health care providers Patients: 836 (273 guidelines only, 254 targeted guidelines, 309 control) all adult patients starting a new episode of care with a study clinician Diagnosis: MDD	Intervention: general guidelines or targeted guidelines. Guidelines based on United Behavioral Health best practice guidelines (based on APA and AHRQ guidelines) Comparator: UC Timing of follow-up: 4 months	Provider behaviors: Mean adjusted adherence rating (subjective) at 4 months* --General dissemination of guidelines vs no dissemination, MD − 0.03 (95% CI not calculable) --Target dissemination vs no dissemination, MD 0.11 (95% CI not calculable) Units of service (indicators of guideline adherence): combined outpatient at 4 months --General dissemination of guidelines vs no dissemination, MD 0.20 (95% CI − 0.65, 1.05) --Target dissemination vs no dissemination, MD − 0.40 (95% CI − 1.25, 0.45) Units of service (indicators of guideline adherence): outpatient medication at 4 months --General dissemination of guidelines vs no dissemination, MD 0.00 (95% CI − 0.43, 0.43) --Target dissemination vs no dissemination, MD − 0.30 (95% CI − 0.74, 0.14) Units of service (indicators of guideline adherence):outpatient psychotherapy at 4 months --General dissemination of guidelines vs no dissemination, MD 0.20 (95% CI − 0.62, 1.02) --Target dissemination vs no dissemination, MD 0.10 (95% CI − 0.75, 0.95) Patient health outcomes: NR
Baker [57] Country: England Setting: primary care office Randomization unit: site	Number of sites: 60 (30 intervention, 30 control) Number of providers: 64 (34 intervention, 30 control) Provider target category: PCP Patients: 402 (210 intervention, 192 control) aged 18 or above attending for their first consultation with new episodes of depression. Diagnosis: depression	Intervention: guideline distribution plus tailored implementation. Guidelines developed from existing guidelines and literature reviews Comparator: other (control group received guidelines but did not receive implementation recommendations) Timing of follow-up: 12 months	Provider behaviors: Antidepressant in therapeutic dose at 12 months OR 1.12 (95% CI 0.99, 1.26) Diagnosis: 3 or more symptoms recorded at 12 months OR 1.25 (95% CI 1.03, 1.53) Reviewed at 3 weeks at 12 months OR 1.04 (95% CI 0.92, 1.18) Suicide risk assessed at diagnosis at 12 months OR 2.51 (95% CI 1.93, 3.26) Those treated are to have two or more follow-up consultations at 12 months OR 1.32 (95% CI 1.07, 1.64) Treated for 4 months at 12 months OR 1.28 (95% CI 1.00, 1.63) Treated with antidepressant or cognitive therapy at 12 months* OR 1.03 (95% CI 0.98, 1.08) Patient health outcomes: BDI < 11 at 16 week at 12 months OR 1.07 (95% CI 0.77, 1.47) BDI < 11 at 4 weeks at 12 months OR 0.83 (95% CI 0.51, 1.34) BDI < 11 at diagnosis at 12 months OR 0.61 (95% CI 0.18, 2.14)
Bosmans [79]; Bijl, 2003 [80] Country: Netherlands Setting: primary care office Randomization unit: Site	Number of sites: 34 (18 intervention, 16 control) Providers: Number NR; PCPs Patients: 145 (70 intervention, 75 control) all consecutive patients 55 years and older visiting their GP Diagnosis: depression (rating scale); Geriatric Depression Scale-15 score of 5 or higher	Intervention: training session based on the Dutch depression guideline Comparator: UC Timing of follow-up: 12 months	Provider behaviors: Received some form of mental health care (antidepressant medication or referral during the follow-up period) at 12 months* --Practitioners training group vs control group, OR 6.64 (95% CI 3.42, 12.90) Patient health outcomes: % (No.) recovered (PRIME-MD) at 12 months OR 0.90 (95% CI 0.61, 1.33) Mean QALYs gained (EQ-5D) at 12 months MD 0.05 (95% CI − 0.02, 0.12) Mean improvement in MADRS score at 12 months MD − 0.60 (95% CI − 3.76, 2.56)
Callahan [81] Country: USA Setting: primary care office Randomization unit: patient	Number of sites: 1 Providers: 103 (number per experimental condition NR) PCPs Patients: 175 (100 intervention, 75 control) aged 60 and older Diagnosis: Depression (rating scale); ≥ 16 on the CES-D and ≥ 15 on the HAM-D	Intervention: Receipt of patient assessment feedback with recommended care. Recommendations based on literature review and expert panel consensus Comparator: UC Timing of follow-up: 6 months	Provider behaviors: Received a depression diagnosis at 6 months OR 2.67 (95% CI 1.36, 5.23) Received a psychiatry referral at 6 months 0.88 (95% CI 0.41, 1.92) Remain on antidepressants at 6 months OR 1.74 (95% CI 1.01, 2.99) Started antidepressants at 6 months OR 3.25 (95% CI 1.41, 7.50) Stopped drugs associated with depression at 6 months* OR 1.05 (95% CI 0.60, 1.82) Patient health outcomes: HAM-D ≤ 10 at 6 months (responder) at 6 months OR 1.08 (95% CI 0.49, 2.40) HAM-D score at 1 month MD 0.20 (95% CI not calculable) HAM-D score at 3 months MD 0.00 (95% CI not calculable) HAM-D score at 6 months MD 0.40 (95% CI not calculable) HAM-D score at 9 months MD 0.60 (95% CI not calculable)
Datto [53] Country: USA Setting: primary care office Randomization unit: site	Number of sites: 35 (17 diseases management, 18 education and guidelines) Providers: 130 (74 disease management, 77 education and guidelines) other general practitioner or clinician Patients: 61 (30 disease management, 31 education and guidelines) Diagnosis: depression (rating scale); CES-D ≥ 16	Intervention: Provider education and distribution of practice guidelines from the AHRQ practice guidelines for major depression in primary care Comparator: Other (education and practice guidelines plus nurse disease management) Timing of follow-up: 16 weeks	Provider behaviors: Clinical adherence, when controlling for symptom improvement at 16 weeks OR 0.18 (95% CI 0.05, 0.67) Clinician adherence through 12 weeks at 16 weeks* OR 0.30 (95% CI 0.08, 1.14) Clinician adherence through 12 weeks, including only patients who required treatment adjustment (*n* = 34) at 16 weeks OR 0.14 (95% CI 0.02, 0.97) Patient adherence through 12 weeks at 16 weeks OR 0.16 (95% CI 0.02, 1.39) Symptom improvement (CES-D < 16) at 16 weeks OR 0.25 (95% CI 0.08, 0.77) Symptom improvement (CES-D < 16) when controlling for clinician adherence, active and passive adherence at 16 weeks OR 0.71 (95% CI 0.10, 1.43) Patient health outcomes: CES-D score at 16 weeks MD 4.50 (95% CI − 0.90, 9.90) Proportion meeting major depression (MINI) at 16 weeks OR 1.92 (95% CI 0.49, 7.69) Proportion of patients below CES-D 11 at follow-up at 16 weeks OR 0.29 (95% CI 0.07, 1.18) Proportion of patients below CES-D 16 at follow-up at 16 weeks OR 0.15 (95% CI 0.04, 0.64) Proportion with at least 50% reduction in CES-D at 16 weeks OR 0.25 (95% CI 0.06, 1.09)
Eccles [87] Country: UK Setting: Primary care trusts Randomization unit: Site	Number of sites: 73 (36 intervention, 37 control) Providers: 266 (128 intervention, 138 control) PCPs Patients: number and description NR Diagnosis: unclear	Intervention: guideline distribution with outreach visits. Guidelines developed by a multidisciplinary panel Comparator: other (guideline distribution only) Timing: 12 months/6 quarters	Provider behaviors: Items prescribed per ASTROPU: MAOIs (mean difference between intervention and control) at 6 quarters MD 0.00 (95% CI − 0.02, 0.02) Items prescribed per ASTROPU: SSRIs (mean difference between intervention and control) at 6 quarters MD 0.03 (95% CI − 0.27, 0.34) Items prescribed per ASTROPU: lofepramine (mean difference between intervention and control) at 6 quarters MD − 0.02 (95% CI − 0.16, 0.11) Items prescribed per ASTROPU: other TCAs (mean difference between intervention and control) at 6 quarters* MD − 0.02 (95% CI − 0.46, 0.42) Number of items prescribed per ASTROPU: MAOIs at 12 months MD − 0.01 (95% CI − 0.04, 0.02) Number of items prescribed per ASTROPU: other TCAs at 12 months MD 0.23 (95% CI − 1.38, 1.84) Number of items prescribed per ASTROPU: SSRIs at 12 months MD 0.41 (95% CI − 0.70, 1.52) Number of items prescribed per ASTROPU lofepramine at 12 months MD 0.05 (95% CI − 0.28, 0.38) Patient health outcomes: NR
Freemantle [83]; Nazareth, 2002 [88] Country: UK Setting: general practices in health authorities (primary care) Randomization unit: site	Number of sites: 12 health authorities paired in groups of 2 randomized to receive 2 of 4 guidelines (3 pairs received antidepressant guidelines, 3 did not). 75 practices (intervention and control Ns NR) Providers: 162 (N per condition NR) PCPs Patients: 11,328 (N per condition not NR). Description NR Diagnosis: unclear	Intervention: outreach visits for providers. Guidelines developed from techniques by the North of England Guidelines Development Project and literature review Comparator: other (practices in 12 health authorities were trained in 2 of 4 guidelines; 3 of 6 health authority pairs did not receive training in antidepressant guideline) Timing of follow-up: 6 months	Provider behaviors: Number of GPs reporting application of content at 6 months* OR 0.61 (95% CI 0.42, 0.91) Patient health outcomes: NR
Gerrity [82] Country: USA Setting: primary care office Randomization unit: provider	Number of sites: NR Providers: 56 (27 intervention, 29 control) PCPs Number of patients: 2 SPs played by 3 actors Diagnosis: unclear	Intervention: depression education training sessions. Guidelines based on AHCPR’s CPG for Depression in Primary Care Comparator: wait-list Timing of follow-up: 6 weeks	Provider behaviors: Physician discussed possibility of depression with “patient 1” at 6 weeks* OR 1.48 (95% CI 1.06, 2.06) Physician discussed possibility of depression with “patient 2” at 6 weeks* OR 1.39 (95% CI 0.86, 2.25) Physician prescribed antidepressants to “patient 1” at 6 weeks OR 1.97 (95% CI 0.85, 4.55) Physician prescribed antidepressants to “patient 2” at 6 weeks OR 1.57 (95% CI 0.89, 2.74) Physician scheduled follow-up within 2 weeks for “patient 1” at 6 weeks OR 2.23 (95% CI 1.26, 3.97) Physician scheduled follow-up within 2 weeks for “patient 2” at 6 weeks OR 2.09 (95% CI 1.19, 3.65) Physician assessed > 5 criteria for major depression in “patient 1” at 6 weeks OR 2.13 (95% CI 1.26, 3.59) Physician assessed > 5 criteria for major depression in “patient 2” at 6 weeks OR 1.70 (95% CI 0.87, 3.31) Physician assessed stresses at home in “patient 1” at 6 weeks OR 1.46 (95% CI 1.09, 1.96) Physician assessed stresses at home in “patient 2” at 6 weeks OR 1.42 (95% CI 0.96, 2.08) Physician assessed suicidal ideation in “patient 1” at 6 weeks OR 13.00 (95% CI 1.82, 92.92) Physician assessed suicidal ideation in “patient 2” at 6 weeks OR 1.04 (95% CI 0.39, 2.77) Patient health outcomes: NR
Goldberg [58]; Horowitz, 1996 [89] Country: USA Setting: primary care office Randomization unit: group practices within primary care clinics	Number of sites: 4 Providers: 95 (allocation to condition reported for 78 providers: academic detailing, 37 academic detailing + CQI team, 23 UC) PCPs Patients: 4995 (allocation to condition reported for 4051 patients: 1073 academic detailing, 1672 academic detailing + CQI team, 1306 UC) age 18 to 75 making clinic visits between February and July 1994 Diagnosis: depression (clinical diagnosis), depression (rating scale)	Intervention: academic detailing and educational sessions based on clinical practice guidelines from the AHCPR Quick Reference Guide for Clinicians Comparator: UC, other (two comparators: (1) usual care and (2) academic detailing plus continuous quality improvement teams (complex system redesign)) Timing of follow-up: 12 months	Provider behaviors: % of eligible known depressives prescribed 1st-generation tricyclics, all clinics at 12 months --Academic detailing vs AD + CQI, OR 0.93 (95% CI 0.79, 1.10) --Academic detailing vs usual care, OR 1.12 (95% CI 0.96, 1.30) % of eligible known depressives prescribed 2nd-generation tricyclics, all clinics at 12 months --Academic detailing vs AD + CQI, OR 0.92 (95% CI 0.72, 1.17) --Academic detailing vs usual care, OR 1.05 (95% CI 0.83, 1.34) % of eligible known depressives prescribed SSRIs, all clinics at 12 months --Academic detailing vs AD + CQI, OR 1.04 (95% CI 0.95, 1.15) --Academic detailing vs usual care, OR 1.02 (95% CI 0.93, 1.12) % of eligible unrecognized depressives prescribed antidepressants, All clinics at 12 months* --Academic detailing vs AD + CQI, OR 1.01 (95% CI 0.62, 1.64) --Academic detailing vs usual care, OR 0.94 (95% CI 0.56, 1.59) Patient health outcomes: SCL score in known depressives, all clinics at 12 months --Academic detailing vs AD + CQI, MD − 0.12 (95% CI not calculable) --Academic detailing vs usual care, MD − 0.09 (95% CI not calculable) SCL score in known depressives, best-case clinic at 12 months --Academic detailing vs AD + CQI, MD − 0.27 (95% CI not calculable) --Academic detailing vs usual care, MD − 0.22 (95% CI not calculable)
Keeley [59] Country: USA Setting: primary care clinics at a federally qualified community health care system Randomization unit: site	Number of sites: 7 (3 motivational interviewing, 4 guideline only) Providers: 21 (10 motivational interviewing, 11 guideline only) PCPs Patients: 171 (85 motivational interviewing, 86 guideline only) 18 years and older Diagnosis: depression (rating scale); PHQ-9 score ≥ 10	Intervention: distribution of practice guideline and recommendations for treatment based on APA’s Practice Guideline for the Treatment of MDD Comparator: other (guidelines plus motivational interviewing training) Timing of follow-up: 24 months	Provider behaviors: Prescription for antidepressant medication at 24 months* OR 0.85 (95% CI 0.43, 1.69) Provider recommendation for physical activity at 24 months OR 0.45 (95% CI 0.20, 1.01) Patient health outcomes: Treatment adherence: days physically active in past week at 24 months MD 1.21 (95% CI 0.37, 2.05) Treatment adherence: filled prescription at 24 months OR 0.79 (95% CI 0.29, 2.08)
Kurian [60]; Trivedi, 2004 [90] Country: USA Setting: primary care office Randomization unit: provider	Number of sites: 3 Providers: 4 (2 intervention, 2 control) PCPs Patients: 55 (32 intervention, 23 control) 18 years and older Diagnosis: MDD, depression (rating scale); ≥ 14 on HDRS-17	Intervention: education plus practice with a computerized support decision system. Guidelines based on APA practice guidelines and consensus expert opinion developed in the Texas Medication Algorithm Project Comparator: other (UC that included initial 1 h training on guidelines) Timing of follow-up: 12 weeks	Provider behaviors: No. of treatment visits at 12 weeks* MD − 1.30 (95% CI − 2.31, − 0.29) Received an adequate antidepressant dose at 12 weeks* OR 1.05 (95% CI 0.72, 1.54) Treatment augmentation (algorithm approved) at 12 weeks OR 0.96 (95% CI 0.24, 3.88) Treatment switch (new antidepressant) at 12 weeks OR 2.52 (95% CI 0.57, 11.02) Patient health outcomes: Rate of remission on HDRS (HDRS ≤ 7) at 12 weeks OR 1.13 (95% CI 0.59, 2.15) Rate of response on HDRS (50% decrease in symptom severity) at 12 weeks OR 0.97 (95% CI 0.63, 1.50) Rate of response on QIDS-SR (≥ 50% decrease in symptom severity) at 12 weeks --Computerized decision support system vs usual care (guidelines and training), OR 1.58 (95% CI 0.70, 3.53)
Lin [78]; Katzelnick, 2000 [91] Country: USA Setting: primary care office Randomization unit: provider	Number of sites: 15 Providers: 109 (53 intervention, 56 UC) PCPs Patients: 124,893 (60,689 intervention, 64,204 UC) Diagnosis: MDD, other depression diagnosis (dysthymic, adjustment, depression NOS) from the HMOs between ages 18–64 whose ambulatory visits were below the top 15th percentile for the prior 2 consecutive years	Intervention: education with group feedback. Guidelines based on DSM-IV diagnostic criteria Comparator: UC Timing of follow-up: 12 months	Provider behaviors: 12 weeks continuous medication at 12 months* OR 0.98 (95% CI 0.81, 1.20) New antidepressant prescriptions/100 visits at 12 months* IRR 1.07 (95% CI 0.90, 1.26) Patient health outcomes: NR
Linden [92] Country: Germany Setting: psychiatry private practice (specialty care) Randomization unit: provider	Number of sites: NR Providers: 103 (20 guidelines plus training, 20 guidelines only, 43 control) mental health care providers Patients: 497 (100 guidelines plus training, 196 guidelines only, 202 control) Diagnosis: unclear	Intervention: receipt of depression guideline alone or with training on WHO depression guidelines and detailed recommendations on patient counseling and management Comparator: UC Timing of follow-up: 12 weeks	Provider behaviors: Adverse drug reactions at 12 weeks --WHO guideline only vs control group, MD − 0.01 (95% CI − 0.04, 0.02) --WHO guideline + training vs control group, MD − 0.01 (95% CI − 0.04, 0.01) Prescribed dosages of mirtazapine, mean mg/day at 12 weeks* --WHO guideline only vs control group, MD − 1.41 (95% CI − 2.87, 0.05) --WHO guideline + training vs control group, MD − 2.38 (95% CI − 4.07, − 0.69) Patient health outcomes: CGI severity at 12 weeks --WHO guideline only vs control group, MD − 0.07 (95% CI − 0.29, 0.15) --WHO guideline + training vs control group, MD − 0.31 (95% CI − 0.57, − 0.05) Patient depression rating at 12 weeks --WHO guideline only vs control group, MD − 1.13 (95% CI − 2.63, 0.37) --WHO guideline + training vs control group, MD − 1.53 (95% CI − 3.33, 0.27) Psychiatrist depression rating at 12 weeks --WHO guideline only vs control group, MD − 2.27 (95% CI − 4.49, − 0.05) --WHO guideline + training vs control group, MD − 3.23 (95% CI − 5.89, − 0.57)
Nilsson [93] Country: Sweden Setting: continuing medical education groups and health care centers (primary care) Randomization unit: provider	Number of sites: 6 health care centers and 3 CME groups Providers: 50 (40 participated: 18 in hypertension group, 8 in peptic ulcer/dyspepsia group, 14 in depression group) other general practitioners or clinicians Patients: 45,982; description NR Diagnosis: unclear	Intervention: pharmacotherapy education group. Guidelines based on literature review and recent national and local recommendations on treatment Comparator: other (delivery of education and feedback regarding non-depression control areas (hypertension and peptic ulcers)) Timing of follow-up: 12 months	Provider behaviors: Fractional prescribing rate: selective serotonin reuptake inhibitors at 12 months MD − 3.80 (95% CI − 12.96, 5.36) Fractional prescribing rate: tricyclic antidepressants at 12 months* MD 2.70 (95% CI − 6.08, 11.48) Prescribed DDDs/1000 patients per year at 12 months* IRR 0.78 (95% CI 0.75, 0.81) Prescribed DDDs/GP at 12 months IRR 1.00 (95% CI 0.97, 1.03) Patient health outcomes: NR
Rollman [61]; Rollman, 2002 [94] Country: USA Setting: academically affiliated primary care practice Randomization unit: provider	Number of sites: 1 Providers: 17 (16 enrolled: 6 active care, 5 passive care, 5 usual care) PCPs Patients: 227 (78 active care, 78 passive care, 71 usual care) Diagnosis: depression (clinical diagnosis)	Intervention: reminders of patients’ depression diagnosis with or without recommendations from AHRQ’s Depression Panel’s Guideline for the treatment of major depression Comparator: UC Timing of follow-up: 6 months	Provider behaviors: # of contacts with any PCP at 6 months --EMR—active care vs EMR—usual care, MD − 0.50 (95% CI not calculable) --EMR—passive care vs EMR—usual care, MD 0.08 (95% CI not calculable) # of contacts with usual PCP at 6 months^1^ --EMR—active care vs EMR—usual care, MD − 0.40 (95% CI not calculable) --EMR—passive care vs EMR—usual care, MD − 0.09 (95% CI not calculable) # of office visits with usual PCP at 6 months --EMR—active care vs EMR—usual care, MD − 0.91 (95% CI not calculable) --EMR—passive care vs EMR—usual care, MD − 0.69 (95% CI not calculable) ≥ 3 contacts with usual PCP at 6 months --EMR—active care vs EMR—usual care, OR 1.58 (95% CI 1.12, 2.21) --EMR—passive care vs EMR—usual care, OR 1.50 (95% CI 1.06, 2.11) Antidepressant medication not offered at 6 months --EMR—active care vs EMR—usual care, OR 1.48 (95% CI 0.93, 2.36) --EMR—passive care vs EMR—usual care, OR 1.45 (95% CI 0.92, 2.29) Antidepressant meds baseline regimen continued without modification at 6 months --EMR—active care vs EMR—usual care, OR 2.43 (95% CI 0.68, 8.76) --EMR—passive care vs EMR—usual care, OR 2.66 (95% CI 0.75, 9.38) Antidepressant meds suggested/prescribed or baseline regimen modified at 6 months --EMR—active care vs EMR—usual care, OR 1.14 (95% CI 0.83, 1.56) --EMR—passive care vs EMR—usual care, OR 1.11 (95% CI 0.81, 1.52) Depression mentioned in ≥ 3 contacts with usual PCP at 6 months --EMR—active care vs EMR—usual care, OR 1.74 (95% CI 0.91, 3.31) --EMR—passive care vs EMR—usual care, OR 1.77 (95% CI 0.94, 3.35) Depression mentioned in any contact with usual PCP at 6 months* --EMR—active care vs EMR—usual care, OR 1.07 (95% CI 0.88, 1.29) --EMR—passive care vs EMR—usual care, OR 1.17 (95% CI 0.99, 1.40) Depression treatment mentioned in ≥ 3 contacts with usual PCP at 6 months --EMR—active care vs EMR—usual care, OR 1.33 (95% CI 0.67, 2.63) --EMR—passive care vs EMR—usual care, OR 1.29 (95% CI 0.65, 2.56) Mental health referral suggested at 6 months --EMR—active care vs EMR—usual care, OR 0.75 (95% CI 0.44, 1.25) --EMR—passive care vs EMR—usual care, OR 1.01 (95% CI 0.64, 1.59) PCP counsels patient for depression at 6 months --EMR—active care vs EMR—usual care, OR 1.19 (95% CI 0.63, 2.25) --EMR—passive care vs EMR—usual care, OR 0.95 (95% CI 0.49, 1.87) Patient health outcomes: HRS-D score at 3 months --EMR—active care vs EMR—usual care, MD − 1.50 (95% CI not calculable) --EMR—passive care vs EMR—usual care, MD 0.50 (95% CI not calculable) HRS-D score at 6 months --EMR—active care vs EMR—usual care, MD − 1.50 (95% CI not calculable) --EMR—Passive care vs EMR—usual care, MD − 1.50 (95% CI not calculable) Recovery rate (HRS-D ≤ 7) at 6 months --EMR—active care vs EMR—usual care, OR 0.98 (95% CI 0.50, 1.91) --EMR—passive care vs EMR—usual care, OR 1.05 (95% CI 0.55, 2.00)
Shirazi [63]; Shirazi, 2009 [95] Country: Iran Setting: primary care office Randomization unit: provider	Number of sites: NR Providers: 192 (96 intervention, 96 control) PCPs Patients: 10 SPs Diagnosis: other depression diagnosis (SPs with depressive symptoms)	Intervention: continuing medical education course tailored toward self-reported stage of change. Guidelines generated by researchers based on literature review Comparator: other (guidelines and education without tailoring to stage of change) Timing of follow-up: 2 months	Provider behaviors: Performance score on appropriate treatment (prescription, lab tests, referrals) at 2 months* --Intervention—large group vs control—large group, MD − 24.00 (95% CI − 44.08, − 3.92) --Intervention—small group vs control—small group, MD − 36.00 (95% CI − 46.76, − 25.24) --Tailored education vs ceducation, MD − 27.00 (95% CI − 35.60, − 18.40) Patient health outcomes: NR
Simon [52] Country: USA Setting: primary care office Randomization unit: patient	Number of sites: 5 Providers: number NR; PCPs Number of patients: 613 patients at participating five primary care clinics who had received new prescriptions for antidepressants, with “new” defined as no antidepressant use in the previous 120 days Diagnosis: depression (clinical diagnosis)	Intervention: receipt of detailed patient report and treatment recommendations based on a computerized algorithm. Guidelines not specified Main dichotomous outcome: patients who receive adequate pharmacotherapy (low dose, > 90 days) Comparator: UC, other (feedback intervention plus care management) Timing of follow-up: 6 months	Provider behaviors: Mental health visits to non-prescribing provider at 6 months --Feedback only vs feedback plus care management, MD − 0.10 (95% CI − 0.93, 0.73) --Feedback only vs usual care, MD 0.22 (95% CI − 1.11, 1.55) Mental health visits to prescribing provider at 6 months* --Feedback only vs feedback plus care management, MD − 0.04 (95% CI − 0.48, 0.40) --Feedback only vs usual care, MD − 0.01 (95% CI − 0.49, 0.47) Patients who receive adequate pharmacotherapy (low dose, > 90 days) at 6 months* --Feedback only vs feedback plus care management, OR 0.91 (95% CI 0.74, 1.13) --Feedback only vs usual care, OR 1.10 (95% CI 0.87, 1.39) Patients who receive adequate pharmacotherapy (moderate dose, > 90 days) at 6 months --Feedback only vs feedback plus care management, OR 0.70 (95% CI 0.50, 0.98) --Feedback only vs usual care, OR 1.17 (95% CI 0.79, 1.73) Patient health outcomes: Depression score at 6 months --Feedback only vs feedback plus care management, MD 0.14 (95% CI not calculable) --Feedback only vs usual care, MD − 0.01 (95% CI not calculable) Major depression by DSM-IV at 6 months --Feedback only vs feedback plus care management, OR 0.53 (95% CI 0.30, 0.94) --Feedback only vs usual care, OR 1.00 (95% CI 0.63, 1.58) Probability of showing 50% decrease in depression score at 6 months --Feedback only vs feedback plus care management, OR 0.79 (95% CI 0.65, 0.95) --Feedback only vs usual care, OR 1.10 (95% CI 0.88, 1.38)
Sinnema [51] Country: Netherlands Setting: general practices (solo practices, group practices or health centers) (primary care) Randomization unit: site	Number of sites: 23 (12 intervention, 11 control) Providers: 46 (23 intervention, 23 control) PCPs Patients: 444 (198 intervention, 246 control) 18 years or older attending participating practices Diagnosis: depression (rating scale); screen positive (≥ 20) on Extended Kessler 10 screening instrument	Intervention: training and consultations from experts with incorporation of personal barriers to guideline implementation on the Dutch College of General Practitioner’s guidelines for depression and anxiety Comparator: other (1-day training from experts on implementing guidelines but no tailored intervention on barriers) Timing of follow-up: 6 months	Provider behaviors: Number of consultations at 6 months* IRR 1.78 (95% CI 1.14, 2.78) Prescribing antidepressants at 6 months* OR 1.07 (95% CI 0.52, 2.19) Referral to specialist mental health services at 6 months OR 1.62 (95% CI 0.72, 3.64) Patient health outcomes: 4DSQ depression at 6 months MD 0.06 (95% CI − 0.52, 0.64) WHODAS II at 6 months MD 1.02 (95% CI − 2.08, 4.12)
van Eijk [65] Country: Netherlands Setting: GPs and pharmacists in peer review groups (primary care) Randomization unit: site	Number of sites: 21 (7 individual intervention, 7 group intervention, 7 control) Providers: 122 (70 GPs and 14 pharmacists in individual intervention, 52 GPs and 9 pharmacists in group intervention, 68 GPs and 13 pharmacists in control) Number of patients: 46,078 people aged 60 years old or over on 1 January 1996 (about 50,000 people) living in the southwest Netherlands health district and insured Diagnosis: unclear	Intervention: group-based on individual-based academic detailing session and review of group- or individual-based performance. Guidelines not specified Comparator: UC Timing of follow-up: 4 months	Provider behaviors: Rate of incident prescriptions of less anticholinergic antidepressants after intervention at 4 months* --Group educational visits vs control group, IRR 1.66 (95% CI 0.97, 2.85) --Individual educational visits vs control group, IRR 2.02 (95% CI 1.24, 3.30) Rate of incident prescriptions of highly anticholinergic antidepressants after intervention: prescriptions/1000 patient years at 4 months --Group educational visits vs control group, IRR 1.79 (95% CI 0.87, 3.57) --Individual educational visits vs control group, IRR 1.47 (95% CI 0.85, 2.56) Patient health outcomes: NR
Worrall [62] Country: Canada Setting: family practice research networks (primary care) Randomization unit: provider	Number of sites: NR Number of providers: 42 PCPs Number of patients: 147, description NR Diagnosis: depression (rating scale)	Intervention: workshop on clinical practice guidelines with follow-up consultations. Guidelines based on Canadian Medical Association’s CPGs Comparator: other (receipt of clinical practice guidelines without education) Timing of follow-up: 6 months	Provider behaviors: Mean no. of office visits per patient at 6 months* MD 0.60 (95% CI − 1.94, 3.14) No. of patients prescribed an antidepressant on first visit at 6 months* OR 1.02 (95% CI 0.91, 1.14) No. of referrals to other mental health professional at 6 months OR 10.53 (95% CI 0.62, 179.01) No. of referrals to psychiatrist at 6 months OR 1.85 (95% CI 0.39, 8.83) Patient health outcomes: CES-D score—patient at 6 months MD 2.80 (95% CI − 1.35, 6.95) CES-D score gain—patient at 6 months MD − 3.80 (95% CI − 8.70, 1.10) No. of patients taking medication at 6-month follow-up at 6 months OR 1.43 (95% CI 0.98, 2.07) No. of patients who took antidepressant for full 6 months at 6 months OR 1.23 (95% CI 0.82, 1.84)
Yawn [64] Country: USA Setting: family medicine research network practices (primary care) Randomization unit: site	Number of sites: 28 (14 intervention, 14 control) Providers: NR (teams) Number of patients: 2343 (1353 intervention, 990 control) women aged at least 18 years, were 5 to 12 weeks’ postpartum Diagnosis: depression (rating scale)	Intervention: education and a set of tools for postpartum depression. Guidelines not specified Comparator: UC Timing of follow-up: 12 months	Provider behaviors: Medication plus counseling at 12 months OR 1.62 (95% CI 1.32, 2.00) Received 2nd call after successful 1st call (women diagnosed with depression) at 12 months* OR 103.48 (95% CI 6.43, 1665.63) Received counseling at 12 months OR 1.82 (95% CI 1.13, 2.93) Treatment with medication at 12 months OR 1.60 (95% CI 1.28, 1.98) Patient health outcomes: Improved PHQ-9, if history of depression at 12 months OR 1.24 (95% CI 0.86, 1.79) Improved PHQ-9, if postpartum depression was diagnosed at 12 months OR 1.10 (95% CI 0.77, 1.56)

Note: * indicates a selected main adherence provider outcome; # number of; ^1^selected main adherence provider outcome not able to be included in analyses due to no reported standard deviation. *APA* American Psychiatric Association; *ASTROPU* Age, Sex and Temporary Resident Originated Prescribing Units; *DDD* defined daily doses; *TCA Tricyclic antidepressants*; *UC* usual care; *NR* not reported; *GP* general practitioner; *PCP* primary care physician/provider; *IRR* incident rate ratio; *OR* odds ratio; *MD* mean difference; *CI* confidence interval; *AD* academic detailing; *PHQ-9* Patient Health Questionnaire-9; *PRIME-MD* PRIMary care Evaluation of Mental Disorders; *WHODAS-II* World Health Organization Disability Assessment Schedule; *4DSQ* The Four-Dimensional Symptom Questionnaire; *HDRS/HAM-D* Hamilton Depression Rating Scale; *CES-D* Center for Epidemiologic Studies Depression Scale

## Results

**Fig. 1:**
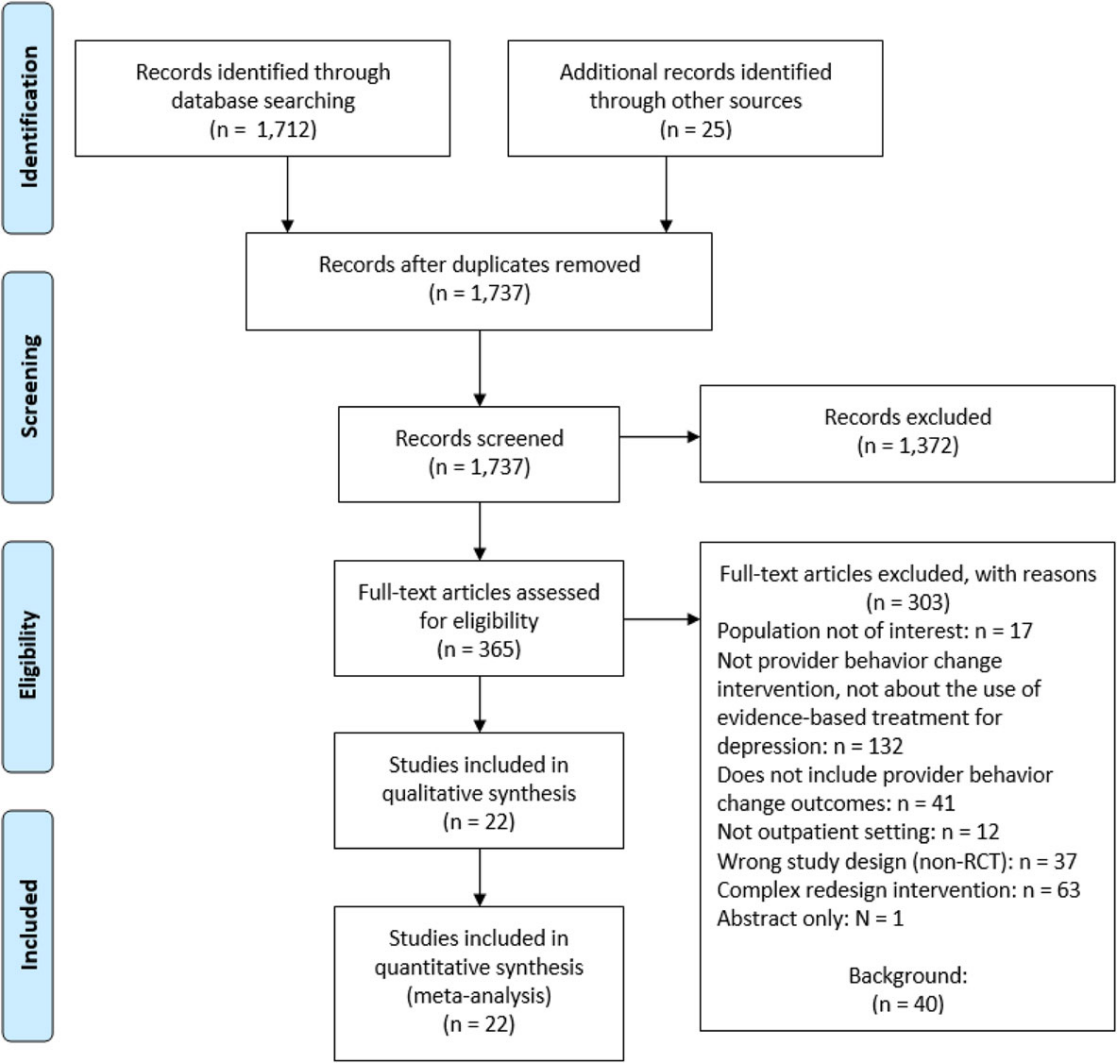
PRISMA flow diagram

The literature search results are documented in a PRISMA [50] literature flow diagram (see Fig. 1). We reviewed 1737 titles and abstracts, and, of these, we reviewed full texts for 365 citations, identifying 22 eligible studies reported in 34 publications. Studies took place in nine countries and included 2149 providers and 239,477 patients. Twenty studies took place in primary care settings, ranging from primary care offices and academically affiliated primary care practices to family medicine research network practices and continuing medical education groups. Two studies took place in specialty care settings: a private psychiatry practice and a managed behavioral health care organization. Two studies included teams of providers and 20 included a single provider only: 16 studies with primary care physicians, two studies with mental health care providers, and two studies with other general practitioners or clinicians. Duration of the interventions, duration of the implementation periods, and the time points of outcome assessment following the end of the implementation phase were all variable. Studies evaluated many types of provider interventions, ranging from simply disseminating depression guidelines to education strategies such as academic detailing and multi-component strategies involving education plus additional components (e.g., reminders or strategies tailored to individual providers) (see Table 1).

The methodological rigor of the included studies was variable; however, all studies were rated high risk of performance bias related to the lack of blinding of intervention providers. It was generally impossible for a provider to be blinded to delivery of the interventions of interest. With respect to the potential for contamination (i.e., both groups sharing material meant for the intervention group), only three out of the 22 studies were judged to be high risk of contamination bias. Half of the included studies described the context and organizational readiness for quality improvement, while the other half did not meet these criteria. Twelve studies met the criterion for reach/penetration domain and described the number of providers or departments that participated in the study compared to the number of available and potentially eligible participants or departments. Only one study addressed the sustainability of the intervention. Details for all critical appraisal domains are shown in Additional file 1: Appendix B.

### Main indications of adherence to depression guidelines

**Fig. 2:**
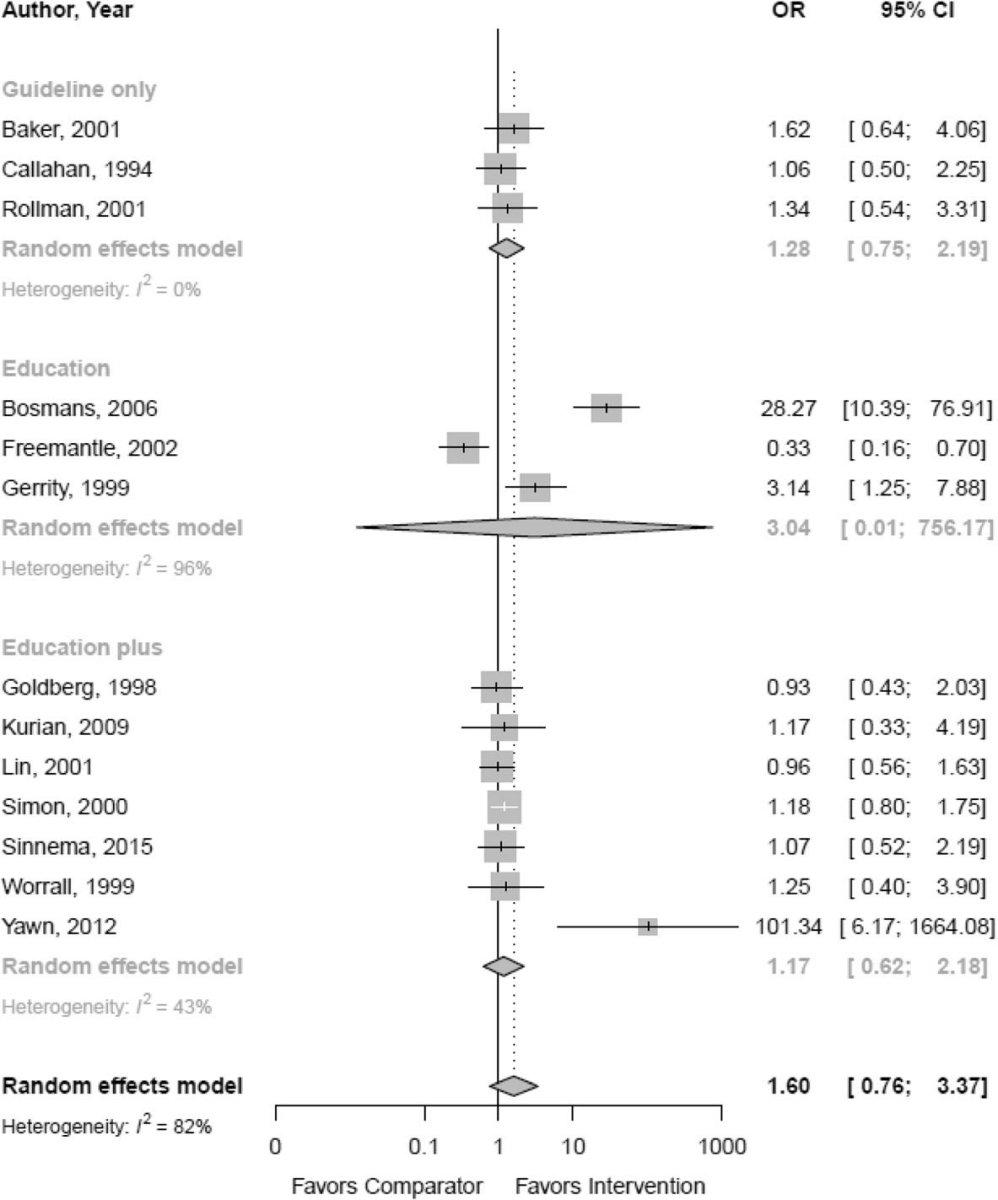
Odds of achieving provider adherence (main indication) compared to usual care practice by intervention type

**Fig. 3:**
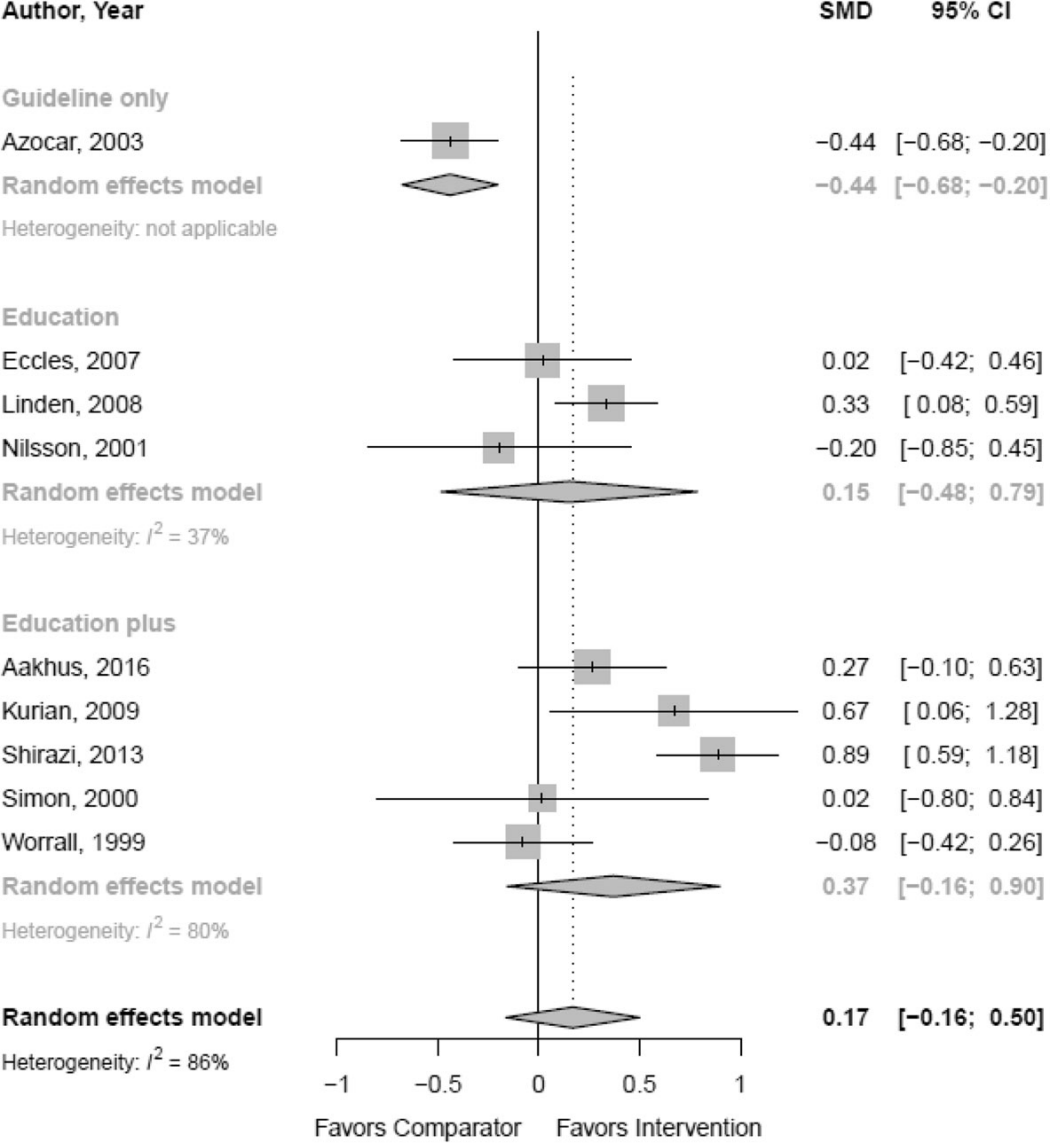
Mean difference in achieved provider adherence (main indication) compared to usual practice by intervention type

Table 1 outlines the findings from individual studies and Table 2 summarizes the evidence for the pooled analyses that utilized the available dichotomous, continuous, and IRR adherence outcomes. Additional file 1: Appendix C includes a summary of findings table with quality of evidence details. Thirteen studies with 3158 participants reported on the odds of achieving provider adherence by comparing a provider intervention to UCP (Fig. 2). Pooled analyses did not indicate a statistically significant difference in the main guideline adherence outcomes across studies (OR 1.60; CI 0.76, 3.37; 13 RCTs; *I*^2^ 82%; moderate QoE). Pooled analyses of nine studies, with 1236 participants, using a continuous outcome also did not show a statistically significant difference compared to UCP (SMD 0.17; CI − 0.16, 0.50; *I*^2^ 86%; low QoE) (Fig. 3). Four studies reporting IRRs also showed no difference between intervention and control groups (IRR 1.16; CI 0.63, 2.15; *I*^2^ 91%; low QoE). However, all analyses showed substantial heterogeneity. Lastly, three studies with 867 participants reported on the odds of achieving provider adherence by comparing a provider intervention to practice redesign efforts; the difference was not statistically significant (OR 0.81; CI 0.30, 2.19; *I*^2^ 20%).

**Table 2:** Summary of findings

Intervention type and outcome measure	Number of RCTs and participants	Reasons for downgrade	Direction and magnitude of relative effect	Grade
Effects of provider intervention on healthcare professional behavior				
Provider intervention vs UCP				
Odds of achieved provider adherence (main indication)	13 RCTs [51, 52, 57, 58, 60–62, 64, 78–83] *N* = 3158	H	Provider interventions not statistically significantly different from comparator groups (OR 1.60; CI 0.76, 3.37)	Moderate
Mean difference in achieved provider adherence (main indication)	9 RCTs [52, 60, 62, 63, 83–87, 92, 93] *N* = 1236	H, DE	Provider interventions not statistically significantly different from comparator groups (SMD 0.17; CI − 0.16, 0.50)	Low
Incidence rate of achieved provider adherence (main indication)	4 RCTs [51, 65, 78, 93] *N* = 63,588	H, IMP	Provider interventions not statistically significantly different from comparator groups (IRR 1.16; CI 0.63, 2.14)	Low
Odds of improved medication prescribing	11 RCTs [51, 52, 57, 58, 60–62, 64, 78, 81, 82] *N* = 4116	H, IMP	Provider interventions statistically significantly different from comparator groups (OR 1.42; CI 1.04, 1.92) favoring the intervention	Low
Mean difference in improved medication prescribing	3 RCTs [86] *N* = 414	DE, IMP	Provider interventions not statistically significantly different from comparator groups (SMD 0.15; CI − 0.48, 0.79)	Low
Incidence rate of improved medication prescribing	3 RCTs [65, 78, 93] *N* = 63,144	H, IMP	Provider interventions not statistically significantly different from comparator group (IRR 1.02; CI 0.44, 2.36)	Low
Odds for increased contact with patients	3 RCTs [61, 64, 82] *N* = 710	H, IMP	Provider interventions not statistically significantly different from comparator groups (OR 6.40; CI 0.13, 322.40)	Low
Mean difference in contact with patients	3 RCTs [52, 60, 62] *N* = 225	IMP	Provider interventions not statistically significantly different from comparator groups (SMD 0.17; CI − 0.84, 1.19)	Moderate
Incidence rate of number of consultations (contact with patients)	1 RCT [51] *N* = 444	S	Provider intervention statistically significantly different from comparator group (IRR 1.78; CI 1.14, 2.78) favoring the intervention	Very low
Odds of general adherence to intervention	6 RCTs [57, 61, 64, 79, 82, 83] *N* = 1375	H, IMP	Provider interventions not statistically significantly different from comparator groups (OR 2.26; CI 0.50, 10.28)	Low
Mean difference in general adherence to intervention	3 RCTs [63, 84, 86] *N* = 597	H, DE, IMP	Provider interventions not statistically significantly different from comparator groups (SMD 0.23; CI − 1.42, 1.89)	Very low
Odds of referral offered to patient	4 RCTs [51, 61, 62, 81] *N* = 896	IMP	Provider interventions not statistically significantly different from comparator groups (OR 1.11; CI 0.33, 3.70)	Moderate
Provider intervention vs practice redesign				
Odds of achieved provider adherence (main indication)	3 RCTs [52, 53, 58] *N* = 867	IMP	Provider interventions not statistically significantly different from comparator groups (OR 0.81; CI 0.30, 2.19)	Moderate
Mean difference in achieved provider adherence (main indication)	1 RCT [52] *N* = 24	S	Provider intervention not statistically significantly different from comparator group (SMD 0.07; CI − 0.73, 0.87)	Low
Odds of improved medication prescribing	2 RCTs [52, 58] *N* = 1738	DE, IMP	Provider interventions not statistically significantly different from comparator groups (OR 0.96; CI 0.18, 5.08)	Low
Mean difference in contact with patients	1 RCT [52] *N* = 24	S	Provider intervention not statistically significantly different from comparator group (SMD 0.07; CI − 0.73, 0.87)	Low
Odds of general adherence to intervention	1 RCT [53] *N* = 61	Poor RoB, IP, S	Provider interventions not statistically significantly different from comparator groups (OR 0.30; CI 0.08, 1.14)	Very low
Provider intervention vs other interventions				
Odds of achieved provider adherence (main indication)	1 RCT [59] *N* = 171	S, IMP, PND	Provider intervention not statistically significantly different from comparator group (OR 0.85; CI 0.43, 1.69)	Very low
Odds of improved medication prescribing	1 RCT [59] *N* = 171	S, IMP, PND	Provider intervention not statistically significantly different from comparator group (OR 0.85; CI 0.43, 1.69)	Very low
Odds of general adherence to intervention	1 RCT [59] *N* = 171	S, IMP, PND	Provider intervention not statistically significantly different from comparator group (OR 0.45; CI 0.20, 1.01)	Very low
Effects by intervention type				
Comparative effectiveness				
Guideline distribution plus implementation recommendations vs guideline distribution alone: odds of achieved provider adherence (main indication)	1 RCT [57] *N* = 378	S, IMP, IP	Provider interventions not statistically significantly different (OR 1.62; CI 0.64, 4.06)	Very low
Guideline distribution and education vs guideline distribution, education, and nurse disease management (system redesign): odds of achieved provider adherence (main indication)	1 RCT [53] *N* = 61	S, IMP, poor RoB, IP	Provider interventions not statistically significantly different (OR 0.30; CI 0.08, 1.14)	Very low
Academic detailing vs academic detailing plus continuous quality improvement: odds of achieved provider adherence (main indication)	1 RCT [58] *N* = 389	S, IMP	Provider interventions not statistically significantly different (OR 1.01; CI 0.48, 2.11)	Very low
Guideline distribution vs guideline distribution and motivational interviewing training: odds for achieved provider adherence (main indication)	1 RCT [59] *N* = 171	S, IMP, PND	Provider interventions not statistically significantly different (OR 0.85; CI 0.43, 1.69)	Very low
Education plus additional training sessions vs education alone: odds for achieved provider adherence (main indication)	1 RCT [60] *N* = 55	S, IMP, PND	Provider interventions not statistically significantly different (OR 1.17; CI 0.33, 4.19)	Very low
Education plus additional training sessions vs education alone: mean difference in achieved provider adherence (main indication)	1 RCT [60] *N* = 55	S, IMP, PND	Provider interventions not statistically significantly different (SMD 0.67; CI 0.06, 1.28)	Very low
Patient-specific treatment recommendations vs recommendations and care management: odds for achieved provider adherence (main indication)	1 RCT [52] *N* = 417	S, IMP	Provider interventions not statistically significantly different (OR 0.85; CI 0.58, 1.25)	Very low
Patient-specific treatment recommendations vs recommendations and care management: mean difference in achieved provider adherence (main indication)	1 RCT [52] *N* = 417	S, IMP	Provider interventions not statistically significantly different (SMD 0.07; CI − 0.73, 0.87).	Very low
Training plus tailored implementation vs training alone: odds for achieved provider adherence (main indication)	1 RCT [51] *N* = 444	S, IMP	Provider interventions not statistically significantly different (OR 1.07; CI 0.52, 2.19).	Very low
Training plus tailored implementation vs training alone: incidence rate for achieved provider adherence (main indication)	1 RCT [51] *N* = 444	S, IMP	Provider interventions statistically significantly different (IRR 1.78; CI 1.14, 2.78), favoring the intervention of training plus tailored implementation	Very low
Guideline distribution plus workshop and consultation vs guideline distribution alone: odds of achieved provider adherence (main indication)	1 RCT [62] *N* = 147	S, IMP	Provider interventions not statistically significantly different (OR 1.25; CI 0.40, 3.90)	Very low
Guideline distribution plus workshop and consultation vs guideline distribution alone: mean difference in achieved provider adherence (main indication)	1 RCT [62] *N* = 147	S, IMP	Provider interventions not statistically significantly different (SMD − 0.08; CI − 0.42, 0.26)	Very low
Education plus other components vs guidelines and education without tailoring to stages of change: mean difference in achieved provider adherence (main indication)	1 RCT [63] *N* = 36	S, IMP	Provider interventions statistically significantly different (SMD 0.89; CI 0.59, 1.18), favoring intervention with education plus other components tailored toward stages to change	Very low
Guideline distribution (passive) vs guideline distribution (active): odds of achieved provider adherence (main indication)	1 RCT [61] *N* = 138	S, IMP, IP	Provider interventions not statistically significantly different (OR 1.76; CI 0.64, 4.86)	Very low
Indirect comparison				
Meta-regression education only vs education plus for odds of achieved provider adherence (main indication)	10 RCTs [51, 52, 58, 60, 62, 64, 78, 79, 82, 83] *N* = 2957	I, IMP	No systematic effect detected (*p* = 0.574)	Very low
Meta-regression education only vs education plus for mean difference in achieved provider adherence (main indication)	8 RCTs [52, 60, 62, 63, 84, 85–87, 92, 93] *N* = 712	I, IMP	No systematic effect detected (*p* = 0.238)	Very low
Meta-regression unidimensional vs multidimensional for odds of achieved provider adherence (main indication)	13 RCTs [52, 57, 58, 60–63, 78–83, 92] *N* = 2953	I, IMP	No systematic effect detected (*p* = 0.707)	Very low
Meta-regression unidimensional vs multidimensional for mean difference in achieved provider adherence (main indication)	9 RCTs [52, 60, 62, 63, 83–87, 92, 93] *N* = 1236	I, IMP	No systematic effect detected (*p* = 0.055)	Very low
Meta-regression unidimensional vs multidimensional for odds of improved medical prescribing	12 RCTs [51, 52, 57–62, 64, 78, 81, 82] *N* = 2678	I, IMP	No systematic effect detected (*p* = 0.317)	Very low
Meta-regression unidimensional vs multidimensional for odds of referral offered to patients	4 RCTs [51, 61, 62, 81] *N* = 896	I, IMP	No systematic effect detected (*p* = 0.195)	Very low
Meta-regression intervention intensity for odds of achieved provider adherence (main indication)	13 RCTs [51, 52, 57, 58, 60–62, 64, 78–83] *N* = 3158	I, IMP	No systematic effect detected (*p* = 0.973)	Very low
Meta-regression intervention intensity for mean difference in achieved provider adherence (main indication)	9 RCTs [52, 60, 62, 63, 83–87, 92, 93] *N* = 1236	I, IMP	The analysis suggested that the intensity of the intervention is associated with the effect size (*p* = 0.033)	Very low
Meta-regression intervention intensity for odds of improved medical prescribing	12 RCTs [51, 52, 57–62, 64, 78, 81, 82] *N* = 2678	I, IMP	No systematic effect detected (*p* = 0.414)	Very low
Meta-regression intervention intensity for odds of general adherence to intervention	8 RCTs [53, 57, 59, 61, 64, 79, 82, 83] *N* = 2411	I, IMP	No systematic effect detected (*p* = 0.542)	Very low
Subgroup analyses by intervention type				
Guideline distribution only: odds of achieved provider adherence (main indication)	3 RCTs [57, 61, 81] *N* = 683	IMP	Provider interventions not statistically significantly different from comparator groups (OR 1.28; CI 0.75, 2.19)	Low
Guideline distribution only: mean difference for achieved provider adherence (main indication)	1 RCT [86] *N* = 281	S, IMP, PND	Provider intervention statistically significantly different from comparator group (SMD − 0.44; CI − 0.68, − 0.20), favoring the comparator	Very low
Guideline distribution only: odds of improved medication prescribing	4 RCTs [57, 59, 61, 81] *N* = 854	H, IMP	Provider interventions not statistically significantly different from comparator groups (OR 1.52; CI 0.60, 3.86)	Low
Guideline distribution only: odds of increased provider contact with patients	1 RCT [61] *N* = 130	S, IMP, IP	Provider intervention statistically significantly different from comparator group (OR 2.71; CI 1.24, 5.94)	Very low
Guideline distribution only: odds of general adherence to intervention	3 RCTs [57, 59, 61] *N* = 679	H, IMP	Provider interventions not statistically significantly different from comparator groups (OR 0.95; CI 0.17, 5.17)	Very low
Education only: odds of achieved provider adherence (main indication)	3 RCTs [79, 82, 83] *N* = 338	H, IMP	Provider interventions not statistically significantly different from comparator groups (OR 3.04; CI 0.01, 756.17)	Low
Education only: mean difference in achieved provider adherence (main indication)	3 RCTs [86] *N* = 414	IMP	Provider interventions not statistically significantly different from comparator groups (SMD 0.15; CI − 0.48, 0.79)	Moderate
Education only: odds of improved medication prescribing	1 RCT [82] *N* = 48	S, IMP	Provider intervention not statistically significantly different from comparator group (OR 2.78; CI 0.80, 9.59)	Very low
Education only: odds of increased provider contact with patients	1 RCT [82]) *N* = 48	S, IMP	Provider intervention statistically significantly different from comparator group (OR 6.42; CI 1.78, 23.18)	Very low
Education only: odds of general adherence to intervention	4 RCTs [53, 79, 82, 83] *N* = 399	H, IMP	Provider interventions not statistically significantly different from comparator groups (OR 2.03; CI 0.06, 73.30)	Very low
Education plus other components: odds for achieved provider adherence (main indication)	7 RCTs [51, 52, 58, 60, 62, 64, 78] *N* = 2090	IMP	Provider interventions not statistically significantly different from comparator groups (OR 1.17; CI 0.62, 2.18)	Moderate
Education plus other components: mean difference in achieved provider adherence (main indication)	5 RCTs [52, 60, 62, 63, 84] *N* = 938	H, IMP	Provider interventions not statistically significantly different from comparator groups (SMD 0.37; CI − 0.16, 0.90)	Low
Education plus other components: odds of improved medical prescribing	7 RCTs [51, 52, 58, 60, 62, 64, 78] *N* = 1710	H	Provider interventions not statistically significantly different from comparator groups (OR 1.21; CI 0.85, 1.71)	Low
Education plus other components: odds of increased provider contact with patients	1 RCT [64] *N* = 483	S, IMP	Provider interventions statistically significantly different from comparator group (OR 101.34; CI 6.17, 1664.08)	Very low
Education plus other components: odds of general adherence to intervention	1 RCT [64] *N* = 482	S	Provider interventions statistically significantly different from comparator group (OR 2.56; CI 1.65, 3.97)	Very low
Effects by provider type				
Meta-regression single provider vs team for odds of achieved provider adherence (main indication)	13 RCTs [51, 52, 57, 58, 60–62, 64, 78–83] *N* = 3158	I, IMP	The analysis suggested that the type of provider is associated with the effect size (*p* = 0.034); however, the analysis is based on only 1 team intervention	Very low
Subgroup analysis by provider type				
Single provider interventions: odds for achieved provider adherence (main indication)	12 RCTs [51, 52, 57, 58, 60–62, 78–83] *N* = 1334	H, IMP	Provider interventions not statistically significantly different from comparator groups (OR 1.42; CI 0.74, 2.73)	Low
Team provider interventions: odds of achieved provider adherence (main indication)	1 RCT [64] *N* = 482	S, IMP	Provider intervention statistically significantly different from comparator group (OR 101.34, CI 6.17, 1664.08), favoring the intervention	Very low
Effect by setting				
Meta-regression primary care vs specialty care setting for mean difference in achieved adherence (main indication)	9 RCTs [52, 60, 62, 63, 83–87, 92, 93] *N* = 1236	I, IMP	No systematic effect detected (*p* = 0.385); however, the analysis is based on only 2 specialty care interventions	Very low
Patient outcomes				
Provider intervention vs UCP				
Mean difference in depression rating scale scores	9 RCTs [51, 52, 58, 61, 62, 79, 81, 84, 92] *N* = 2196	DE	Provider interventions not statistically significantly different from comparator groups (SMD − 0.06; CI − 0.14, 0.01)	Moderate
Odds of depression treatment response	6 RCTs [52, 57, 60, 61, 64, 81] *N* = 1312	DE	Provider interventions statistically significantly different from comparator groups (OR 1.12; CI 1.04, 1.21) favoring the intervention	Moderate
Odds of depression recovery	6 RCTs [52, 57, 60, 61, 79, 81] *N* = 1274	DE	Provider interventions not statistically significantly different from comparator groups (OR 1.02; CI 0.91, 1.15)	Moderate
Odds of depression treatment adherence	2 RCTs [62, 84] *N* = 281	IMP	Provider interventions not statistically significantly different from comparator groups (OR 1.52; CI 0.70, 3.31)	Moderate
Provider intervention vs system redesign				
Mean difference in depression rating scale scores	3 RCTs [52, 53, 58] *N* = 861	IMP	Provider interventions not statistically significantly different from comparator groups (SMD 0.09; CI − 0.48, 0.67)	Moderate
Odds of depression treatment response	2 RCTs [52, 53] *N* = 478	IMP	Provider interventions not statistically significantly different from comparator groups (OR 0.53; CI 0.01, 40.38)	Moderate
Odds of depression recovery	2 RCTs [52, 53] *N* = 478	IMP	Provider interventions not statistically significantly different from comparator groups (OR 0.41; CI 0.01, 17.89)	Moderate
Odds of depression treatment adherence	1 RCT [53] *N* = 61	S	Provider interventions not statistically significantly different from comparator groups (OR 0.16; CI 0.02, 1.39)	Very low
Provider intervention vs other interventions				
Odds of depression treatment adherence	1 RCT [59] *N* = 171	S, IMP	Provider intervention not statistically significantly different from motivational interviewing (OR 0.79; CI 0.30, 2.08)	Very low
Mean difference in treatment adherence	1 RCT [59] *N* = 171	S	Provider intervention not statistically significantly different from motivational interviewing (SMD − 0.43; CI − 0.76, − 0.11)	Very low

Notes: For GRADE, the following were consider: *study limitations* (low, medium, or high risk of bias), *indirectness* (direct or indirect), *inconsistency* (consistent, inconsistent, or unknown), *imprecision* (precise or imprecise), and *reporting bias* (likely present or not applicable). *H* heterogeneity downgrade; *DE* direction of effects downgrade; *S* single study downgrade; *I* indirect effects downgrade; *IMP* imprecision downgrade; *IRR* incidence rate ratio; *OR* odds ratio; SMD standardized mean difference; *UCP* usual care practice; *vs* versus; *Poor RoB* study rated with poor quality, *PND* power not discussed, *IP* insufficient power,

### Medication prescribing

Eleven studies with 4116 participants reported on the odds of improved medication prescribing. The pooled analysis indicated a statistically significant intervention effect favoring the intervention compared to UCP (OR 1.42; CI 1.04, 1.92; *I*^2^ 53%). Intervention providers were more likely to prescribe according to clinical practice guidelines. Three studies with 414 participants did not indicate a statistically significant effect in reporting on a continuous outcome (SMD 0.15; CI − 0.48, 0.79; *I*^2^ 37%). Three studies reporting on IRRs did not show a difference between intervention and control groups (IRR 1.02; CI 0.44, 2.36; *I*^2^ 90%). Two studies with 1738 participants with a practice redesign comparator showed conflicting results, and pooled results were not statistically significant (OR 0.96; CI 0.18, 5.08; *I*^2^ 0%). Additional file 1: Appendix C contains further details of these analyses and analyses for all other individual outcomes summarize below.

### Contact with patients

Three studies with 710 participants reported on the odds of increased patient contacts in studies comparing a provider intervention with UCP. The pooled analysis showed no statistically significant difference between intervention and control groups (OR 6.40; CI 0.13, 322.40; *I*^2^ 75%). Similarly, three studies comparing a provider intervention with UCP, with 225 participants, also did not report a statistically significant difference using continuous outcomes (SMD 0.17; CI − 0.84, 1.19; *I*^2^ 56%). One study [51] with 444 participants reported IRR data on the number of provider consultations at 6-month follow-up. This study reported a significant effect favoring the intervention, which consisted of training on guidelines and consultations from experts to address personal barriers to implementing guidelines (IRR 1.78; CI 1.14, 2.78). The only study reporting outcomes on contact with patients compared to a system redesign effort was a small study of 24 participants [52]; the study found no difference for the outcome (SMD 0.07; CI − 0.73, 0.87). Providers in this study intervention group received a detailed report on their patients that contained treatment recommendations based on a computerized algorithm, while the system redesign group received this feedback enhanced with care management of patients by care managers who helped to implement the physicians’ recommendations.

### Specific intervention adherence

Indicators of this outcome were variable and included whether or not patients were treated using a specific treatment from the guidelines, the number of providers adhering to the specifications of the guidelines, and a performance score received by the providers on whether or not they offered appropriate treatment specified by the guidelines. Six studies with 1375 participants reported on the odds of adherence compared to UCP. The pooled analysis for dichotomous outcomes did not indicate a statistically significant intervention effect (OR 2.26; CI 0.50, 10.28; *I*^2^ 90%). Three studies with 597 participants reporting on a continuous outcome also did not indicate a statistically significant difference (SMD 0.23; CI − 1.42, 1.89; *I*^2^ 96%). No study reported on IRR outcomes. One small, high risk of bias study [53] reported on a measure of intervention adherence where a provider intervention was compared to practice redesign. There was no statistically significant difference between comparator and intervention, which offered education and distribution of practice guidelines to primary care providers (OR 0.30; CI 0.08, 1.14).

### Referral offered to patients

Four studies with 896 participants and a UCP comparator reported on the odds of improved referral offered to patients by providers. The pooled analysis did not indicate a systematic intervention effect (OR 1.11; CI 0.33, 3.70; *I*^2^ 41%). No identified studies reported on continuous or IRR outcomes on referral outcomes compared to practice redesign.

### Patient outcomes

We identified 14 studies that reported patient outcomes. Specifically, these studies reported changes in depression rating scale scores, depression treatment response (i.e., proportion of patients with improvement, including remission), depression recovery (i.e., proportion of patients in remission/not meeting depression criteria at follow-up), and treatment adherence (e.g., medication adherence). Nine studies with 2196 participants reported on the mean difference in patient depression rating scales, such as the Hamilton Rating Scale for Depression [54] or the Center for Epidemiologic Studies Depression Scale [55]. A pooled analysis comparing provider interventions to UCP did not indicate a difference in patient outcomes associated with the intervention (SMD − 0.06; CI − 0.14, 0.01; *I*^2^ 0%). Six studies with 1312 participants reported on depression treatment response (e.g., a score less than 11 on the Beck Depression Inventory [56]). The pooled analysis indicated a statistically significant effect favoring the provider interventions when compared to UCP (OR 1.12; CI 1.04, 1.21; *I*^2^ 0%). Six studies with 1274 participants reported on patient recovery from depression; the pooled analysis did not indicate a statistically significant effect of the provider intervention when compared to UCP (OR 1.02; CI 0.91, 1.15; *I*^2^ 0%). Two studies involving 281 participants and a UCP comparator reported on patient treatment adherence as indicated by the number or proportion of patients who took prescribed antidepressants as indicated. The pooled analysis did not indicate a statistically significant difference between the provider intervention and UCP (OR 1.52; CI 0.70, 3.31; *I*^2^ 0%). Lastly, three studies with 861 participants reported on the mean difference in patient depression rating scales as compared to practice redesign efforts. The pooled analysis did not indicate a statistically significant difference between the provider intervention and practice redesign (SMD 0.09; CI − 0.48, 0.67; *I*^2^ 52%). Two studies with 478 participants and a practice redesign comparator reported on depression treatment response; the analysis did not indicate a statistically significant difference (OR 0.53; CI 0.01, 40.38; *I*^2^ 26%).

### Findings by type of intervention

Ten studies provided direct comparative effectiveness results utilizing the main indications of adherence to depression guidelines outcomes [51–53, 57–63] (see Additional file 1: Appendix C) of which two reported significant differences. One study reporting a significant difference [51], involving 444 participants, compared provider training in guidelines plus tailored implementation to provider training alone. This study found no statistically significant difference in odds for achieving provider adherence at 6-month follow-up (OR 1.09; CI 0.62, 1.91) but did find a significant effect favoring the group that received provider training in guidelines plus tailored implementation for the incidence risk for achieving provider adherence (IRR 0.85; CI 0.43, 1.69). The other study reporting a significant difference [63], involving 389 participants, found a statistically significant mean difference between intervention and comparator groups (SMD 0.89; CI 0.59, 1.18). The intervention consisted of a 2-day continuing medical education course focused on treatment and differential diagnosis of depression disorders. Providers were assigned to groups in which the education component was tailored to the providers’ self-reported stage of change. The comparator group also received education on treatment and diagnosis of depression disorders (including the same 2-day continuing medical education course), but the education was not tailored to the providers’ stage of change.

Indirect comparisons using meta-regression determined whether within the range of eligible provider interventions, those that combined education with other components, such as tailored implementation strategies, reported better results than education-only interventions. The analyses did not indicate that interventions classified as education only systematically reported different effects than interventions with additional components (dichotomous outcomes *p* = 0.574, continuous outcomes *p* = 0.238). We also compared unidimensional and multidimensional interventions. We found no statistically significant effect for dichotomous outcomes (*p* = 0.707), but the equivalent analysis for studies reporting continuous outcomes approached statistical significance (*p* = 0.055). To explore this finding further, we rated the intensity of the intervention on a 3-point scale. A meta-regression for the dichotomous adherence outcome did not show an effect (*p* = 0.973); however, the analysis of the continuous adherence outcome suggested that the intensity of the intervention was associated with the effect size (i.e., the greater the intensity, the greater the adherence; *p* = 0.033). The analysis should be interpreted with caution because of the small number of studies contributing to individual intensity rating categories.

For subgroup analyses, we stratified the included studies by those distributing guidelines to providers, those with education interventions, and those with more complex interventions that included, for example, an education component in addition to exploring and helping providers overcome barriers to guideline implementation. Figures 2 and 3 show the individual study results for the dichotomous and continuous main indication of adherence outcomes within these broad subgroups. All three subgroups still reported no statistically significant differences between the intervention and the comparator groups. There was no statistically significant intervention effect in studies that simply distributed treatment guidelines (OR 1.28; CI 0.75, 2.19; 3 RCTs; *I*^2^ 0%; SMD − 0.44; CI − 0.68, − 0.20; 1 RCT). Three studies evaluated an education intervention and showed conflicting results (OR 3.04; CI 0.01, 756.17; *I*^2^ 95. SMD 0.15; CI − 0.48, 0.79; *I*^2^ 37%). The pooled analysis of studies of education plus other components also did not indicate a statistically significant intervention effect (OR 1.17; CI 0.62, 2.18; *I*^2^ 44%, 7 RCTs. SMD 0.37; CI − 0.16, 0.90; *I*^2^ 80%; 5 RCTs).

### Findings by provider target

We did not identify any studies directly comparing effects for different types of healthcare providers. We indirectly compared the 20 interventions that targeted single providers and the two that targeted teams. For the dichotomous adherence outcome, a meta-regression indicated that the intervention effect systematically varied by the type of provider targeted (*p* = 0.034); yet, the analysis should be interpreted with caution because only one of the team studies contributed data to this [64]. The effect was not replicated in an analysis based on IRR data that compared the other team intervention [65] with the three sole provider interventions that had count outcomes (*p* = 0.352).

For subgroup analyses, we stratified the results by interventions on single providers versus teams of providers (see Additional file 1: Appendix C). Pooled analyses of studies of interventions that targeted single providers did not report a statistically significant intervention effect on the main adherence outcome (OR 1.42; CI 0.74, 2.73; 12 RCTs; *I*^2^ 80%). One study [64] compared a team intervention to a control, and the effect for provider follow-up with patients was significant in favor of the intervention group at 12-month follow-up (OR 101.34, CI 6.17, 1664.08). Given the wide confidence interval, we looked at another main adherence outcome (i.e., whether the patient received medication plus counseling) and similarly found an effect favoring the team intervention (OR 1.50; CI 0.83, 2.73).

### Findings by setting

We did not identify any studies directly comparing the effects of the setting. To assess whether effects varied by setting, we compared two studies conducted in specialty care settings with 20 conducted in primary care settings (see Additional file 1: Appendix C). A meta-regression on the main continuous adherence outcome did not suggest any systematic effects of the setting (*p* = 0.385), but the result should be interpreted with caution as only two studies provided data on specialty care settings.

## Discussion

This systematic review compiles research evidence on the effects of healthcare provider interventions on adherence to guidelines or guideline-concordant behavior for depression treatment. We excluded system redesign efforts as interventions for our purposes (e.g., collaborative care where infrastructures are re-organized) and targeted studies that included provider behavior change outcomes. Our findings provide little support for the effectiveness of currently tested provider education or dissemination interventions on provider adherence to depression treatment guidelines; however, there was some evidence that provider interventions improved the outcomes of medication prescribing and patient depression treatment response. Results also suggested that some interventions that were tailored to providers’ needs and that went beyond simply distributing guidelines to providers may improve provider behavior and promote guideline adherence.

Our findings are important for several reasons. First, it is important for healthcare systems to know whether the approaches identified in our review, all of which are less costly to implement than major systems change interventions, such as collaborative care, can change provider behavior. Second, few, if any, interventions including collaborative care for depression are undertaken without an education-focused component. This study can help focus efforts to better evaluate and improve this component. Third, provider education can be a critical step in promoting readiness to improve depression care and, if carried out effectively, may often be the best first implementation step in depression care improvement initiatives. This study provides a foundation for further development of provider education and dissemination methods for improving depression care.

While there is a substantial body of evidence on provider interventions in terms of research volume, it is noteworthy that we evaluated many unique interventions, ranging from the simple distribution of guidelines to education strategies only and further to education that involved multiple follow-up components and trainings. No two studies reported on the same intervention and comparator which limits comparative analyses. We assessed whether educational interventions alone can change provider behavior in clinical practice and, in line with existing reviews [30, 31], our analyses did not find significant effects. Indirect comparisons across the identified studies to detect effect modifiers indicated that more complex interventions (i.e., those with provider education plus additional components and implementation strategies such as tailoring training to address personal implementation barriers) may be associated with more favorable outcomes. However, we did not identify subgroups of interventions that were consistently associated with significant changes in provider behavior. The individual successful approaches observed for main adherence outcomes have not been investigated in more than one study, and findings have not been replicated across independent researcher groups. More studies are needed that attempt to isolate specific provider interventions employed either within system redesigns or in studies that evaluate provider interventions specifically. As described, the methodological rigor in included research studies varied, but none of our analyses were exclusively based on poor quality studies. We also found no indication of publication bias. In addition, because the outcomes we were interested in were often not the primary outcome of the research studies (e.g., studies were often interested in the impact on patients), we are also more confident that estimates in our review are less likely to be affected by publication bias. Still, given the diversity of the interventions evaluated in individual studies, the heterogeneity in results, and results based on single studies, often with imprecision in effect estimates and with follow-up periods of 1 year or less, the quality of evidence remains very limited.

Though we did not identify statistically significant differences for the main adherence outcomes across the interventions compared to UCP, analyses showed heterogeneity and wide confidence intervals that support the possibility of a large range of potential intervention effects. A pooled analysis of 11 RCTs indicated increased odds of improved medication prescribing, which is arguably the aspect of depression care most under the healthcare providers’ control. There was no indication of publication bias; however, we detected considerable heterogeneity and not all studies favored the intervention. Therefore, we believe the finding to have low quality of evidence. Furthermore, no statistically significant difference emerged in an analysis for improved medication prescribing utilizing a continuous operationalization of the outcome. One study [51] showed an increased rate of contact with patients following training and consultations from experts on guidelines that incorporated personal barriers to implementing the guidelines, compared to UCP. However, the result is based on a single study, and therefore, we have limited confidence in this finding. No other specific provider behavior outcome was found to be significant for provider interventions compared to any comparator.

Due to the small number of studies reporting team interventions or interventions in specialty care, we did not find statistically robust evidence that intervention effects varied by targeted provider group or setting. Our review findings suggest that interventions targeting multidisciplinary team members are more effective than interventions targeting only healthcare providers directly, but additional research studies are needed to confirm this finding. Given the lack of studies in specialty care settings, more studies conducted in specialty care settings are also needed to understand how evidence-based interventions can best be adopted by providers outside of psychiatric research settings.

The review findings for effects on patient health were mixed. Although depression treatment response improved across the identified intervention, we did not find significant effects for other patient outcomes such as depression rating scale scores, depression recovery, or treatment adherence. The findings for patient outcomes should be interpreted in context because we restricted the review to studies that reported on provider outcomes. Prior reviews have evaluated how provider interventions affect patient outcomes and have concluded that multi-faceted and system redesign approaches were more effective in improving patient outcomes than simpler or single component interventions, such as distribution of guidelines and education alone [30, 31]. Yet, our review set out to identify interventions that can be implemented in healthcare organizations without practice redesign efforts and more studies are needed that report on both provider behavior change outcomes and patient outcomes in order to better understand whether provider behavior is affected by the intervention and if the change in provider behavior is ultimately affecting patient health.

This review has several strengths, including an a priori research design, duplicate reviewer study selection and data abstraction of study information, a thoughtful and thorough literature search not restricted to a small set of known interventions, detailed critical appraisal, and comprehensive quality of evidence assessments used to formulate review conclusions. Yet, limitations remain. First, our review documents results of RCTs, a robust study design that allows confident evidence statements. Evidence from RCTs and pre-post studies cannot easily be combined methodologically, and we chose to restrict to RCTs. All forest plots are based on studies with concurrent control groups randomly assigned to an intervention condition. Nonetheless, we acknowledge that some authors [66] and the Cochrane Effective Practice and Organisation of Care Group have recommended other study designs such as controlled before-after studies, in addition to RCTs, when evaluating organizational interventions. However, we were specifically interested in the presence and absence of this strong and universally accepted study design to document the state of evidence for provider interventions. An exploratory search for non-RCT literature indicated that results reported with other study designs appear to be similarly mixed in non-randomized controlled studies, time-series, pre-post studies, and cohort studies [67–73]. Second, by including only studies that measure provider behavior and outcomes, we were able to judge whether the intervention is having the intended effects on the target of the intervention (i.e., the providers). Nonetheless, this restriction excluded a large number of existing research studies that do not report on provider behavior. Third, to be included, studies had to report on depression treatment. Effects on improving recognition, screening, or diagnosis of patients or on increasing referral behavior to specialty mental health care settings should be assessed in future systematic reviews. Lastly, the individual interventions and promoted depression practice guidelines varied across studies. Our review included studies using prominent guidelines such as the Agency for Health Research and Quality (AHRQ) treatment guidelines for depression in primary care [74], in addition to studies using treatment guidelines for which we could not verify whether the guidelines were evidence-based. The specific guidelines utilized within the interventions themselves varied and ranged from the American Psychiatric Association Practice Guidelines for the Treatment of Psychiatric Disorders to the Dutch College of General Practitioners’ Practice Guideline for Depression to the Agency for Health Care Policy and Research Practice Guidelines for Depression [75–77]. Many of the studies did not specify in detail how lengthy or how much of a time commitment the guidelines were for providers, which could have accounted for the provider change behaviors findings described within the individual included studies. Some standardization across studies regarding which and how guidelines were utilized in practice appears needed. Such standardization could help account for confounding factors in research studies, but the field may also benefit from a single source of information on best treatment practices for depression.

## Conclusions and future directions

Fourteen years ago, Gilbody and colleagues [30] reviewed organizational and educational interventions targeted at primary care providers treating depressed patients. Authors concluded that effective strategies to improve depression management in this setting were multi-faceted (e.g., system redesign approaches including screening for depression, providing education to patients, and realignment of professional roles in an organization). Sikorski and colleagues [31] similarly concluded that provider training alone does not seem to improve depression care. Our review shows that, despite new research, provider interventions focused primarily on guideline distribution or education only are unlikely to be effective in the absence of additional components. Our review did not identify subgroups or categories of interventions that were consistently associated with increased adherence to depression guidelines or guideline-concordant practices. These findings underscore the need for further research to better understand how to effectively change provider behavior in differential care settings without organizational redesigns. Innovations are needed to support healthcare organizations that want to improve guideline adherence but do not intend to invest in efforts to restructure how care is delivered. Research on provider interventions should be supported by a framework that allows for a more structured assessment to identify successful intervention approaches and the effects of individual intervention components.

## Additional file

Additional file 1: **Appendix A:** Search strategy. **Appendix B:** Critical appraisal ratings using the Cochrane Risk of Bias tool and the QI-MQCS. **Appendix C:** Detailed quality of evidence and summary of findings. (DOCX 707 kb)
