# Supplementary material for: Elusive search for effective provider interventions: a systematic review of provider interventions to increase adherence to evidence-based treatment for depression
Source: Implement Sci. 2018 Jul 20;13:99. doi: 10.1186/s13012-018-0788-8 (PMC6053754; doi:10.1186/s13012-018-0788-8)
Supplement: Supplementary file 1 — Appendix A: Search strategy. Appendix B: Critical appraisal ratings using the Cochrane Risk of Bias tool and the QI-MQCS. Appendix C: Detailed quality of evidence and summary of findings. (DOCX 707 kb) [file 13012_2018_788_MOESM1_ESM.docx]

**Appendix A: Search Strategy**

MEDLINE OVID

*Search 1: initial on 12/15/2016 and update on 8/25/2017*

| 1 | (Depress$ or dysthymia or mood dysregulation or premenstrual dysphoric).tw. or depressive disorder/ | 418737 |
| --- | --- | --- |
| 2 | ((quality and improv* and intervention$) or knowledge translation or Implement* or research to practice).tw. | 426871 |
| 3 | (evidence-based or guideline$ or care protocol or treatment recommendation or recommended treatment or appropriate care).tw. | 349099 |
| 4 | 2 and 3 | 44963 |
| 5 | 1 and 4 | 1450 |
| 6 | exp *education,continuing/ | 34995 |
| 7 | ((education$ adj3 (program$ or intervention? or meeting? or session? or strateg$ or workshop? or visit?)) or disease management program).tw. | 69154 |
| 8 | (behavio?r$ adj2 intervention?).tw. | 12106 |
| 9 | pamphlets/ | 3684 |
| 10 | (leaflet? or booklet? or poster? or pamphlet?).tw. | 32685 |
| 11 | ((written or printed or oral) adj information).tw. | 1973 |
| 12 | (information$ adj2 campaign).tw. | 434 |
| 13 | (education$ adj1 (method? or material?)).tw. | 6382 |
| 14 | *advance directives/ | 3397 |
| 15 | outreach.tw. | 11119 |
| 16 | (((opinion or education$ or influential) adj1 leader?) or ((opinion or education$ or influential) adj1 champion)).tw. | 1414 |
| 17 | facilitator?.tw. | 19482 |
| 18 | (academic detailing or train the trainer).tw. | 961 |
| 19 | consensus conference?.tw. | 5102 |
| 20 | (consultation and supervision and coaching).ti,ab. | 2 |
| 21 | (Depression education or Continuing education or competence training or learning collaborative).tw. | 460 |
| 22 | *guideline adherence/ | 13016 |
| 23 | practice guideline?.tw. | 20593 |
| 24 | ((guideline? adj2 (compl$ or implement$ or introduc$ or issu$ or impact or effect$ or disseminat$ or distribut$ or learn or adopt$ or rollout or roll-out)) and depression management).tw. | 2 |
| 25 | (toolkit? adj2 (compl$ or implement$ or introduc$ or issu$ or impact or effect$ or disseminat$ or distribut$ or learn or adopt$ or rollout or roll-out)).tw. | 163 |
| 26 | (evidence-based adj2 (compl$ or implement$ or introduc$ or issu$ or impact or effect$ or disseminat$ or distribut$ or learn or adopt$ or rollout or roll-out)).tw. | 6137 |
| 27 | ((compl$ or effect$ or impact or evaluat$ or introduc$ or compar$) adj2 training program$).tw. | 1505 |
| 28 | *reminder systems/ | 1865 |
| 29 | (reminder? or clinical support tool).tw. | 10213 |
| 30 | (recall adj2 system$).tw. | 485 |
| 31 | (prompter? or prompting).tw. | 7109 |
| 32 | algorithm?.tw. | 196983 |
| 33 | *feedback/ or feedback.tw. | 115046 |
| 34 | chart review$.tw. | 33562 |
| 35 | ((effect? or impact or records or chart?) adj2 audit).tw. | 993 |
| 36 | exp *reimbursement mechanisms/ | 19137 |
| 37 | fee for service.tw. | 4670 |
| 38 | or/6-37 | 599092 |
| 39 | (clinician? or practitioner? or pharmacist? or provider? or physician? or doctor? or counselor? or therapist? or psycholog$ or psychiatr$).ti,ab. | 1211869 |
| 40 | (nurse adj (rehabilitator? or clinician? or practitioner? or provider?)).ti,ab. | 10641 |
| 41 | (patient care team? or practice team?).ti,ab. | 779 |
| 42 | exp *patient care planning/ | 27965 |
| 43 | (integrat$ adj2 (care or service?)).tw. | 11221 |
| 44 | (care adj2 (coordinat$ or program$ or continuity)).tw. | 26186 |
| 45 | (case adj1 management).tw. | 9906 |
| 46 | physician's practice patterns/ | 51729 |
| 47 | quality assurance.tw. | 23063 |
| 48 | *process assessment/ [health care] | 2123 |
| 49 | *program evaluation/ | 9530 |
| 50 | exp *"Referral and Consultation"/ and "consultation"/ | 23825 |
| 51 | *drug therapy,computer assisted/ | 1222 |
| 52 | *health maintenance organizations/ | 9524 |
| 53 | (managed care or general practice).tw. | 50051 |
| 54 | or/39-53 | 1360891 |
| 55 | 38 and 54 | 109749 |
| 56 | 1 and 55 | 5164 |
| 57 | (quality and ((continuous$ or total) adj5 (manag$ or improv$))).tw. | 7616 |
| 58 | ((continuous$ or total) and (quality adj3 (manag$ or improv$))).tw. | 24685 |
| 59 | (CQI or TQM).tw. | 1501 |
| 60 | total quality management/ | 12568 |
| 61 | quality manag$.tw. | 5645 |
| 62 | ((process or processes or system or systems) adj3 (improving or improvement or improve or redesign$)).tw. | 22180 |
| 63 | model for improvement.tw. | 407 |
| 64 | ((improvement or QI or quality assurance or QA) adj5 (team? or microsystem? or cycle?)).tw. | 2404 |
| 65 | (PDSA or PDCA or TQIS or plan do study or plan do check).tw. | 1020 |
| 66 | ((shewhart or shewart or deming) adj3 (cycle or method)).tw. | 78 |
| 67 | (breakthrough adj3 (series or project or collaborative?)).tw. | 195 |
| 68 | (lean adj (approach or management or method? or methodology or thinking or enterpri#e or practice or philosophy or principles)).tw. | 392 |
| 69 | six sigma.tw. | 505 |
| 70 | or/57-69 | 64822 |
| 71 | 1 and 70 | 1388 |
| 72 | 5 or 56 or 71 | 7424 |
| 73 | Randomized controlled trial.pt. or (random$.tw. and (publisher or pubmed-not-medline or in process).st.) | 677399 |
| 74 | 72 and 73 | 1642 |
| 75 | (mouse or mice or rats or dogs).ti. | 880883 |
| 76 | 74 not 75 | 1642 |
| 77 | humans/ or (publisher or pubmed-not-medline or in process).st. | 20007414 |
| 78 | 76 and 77 | 1639 |
| 79 | limit 78 to english language | 1598 |
| 80 | 79 | 1598 |
| 81 | limit 80 to yr="2017" | 130 |
| 82 | 79 | 1598 |
| 83 | limit 82 to yr="2016" | 158 |
| 84 | from 83 keep 25, 46-47, 56 | 4 |
| 85 | 81 or 84 | 134 |

### Search 2 (adding behavioral change techniques): initial on 2/10/2017 and update on 8/28/2017

| 1 | Persuasion.tw. or persuasive communication/ | 4400 |
| --- | --- | --- |
| 2 | (incentivise or incentivize or incentivization or incentivisation or incentive*).tw. | 25041 |
| 3 | (environmental adj2 restructuring).tw. | 26 |
| 4 | (behavioral* modeling or behavioural* modeling).tw. | 49 |
| 5 | action planning.tw. | 875 |
| 6 | (provider behaviour or provider behavior).tw. | 344 |
| 7 | (behavi?r* adj substitution).tw. | 14 |
| 8 | (behavi?r* adj2 contract).tw. | 50 |
| 9 | cue signaling.tw. | 48 |
| 10 | (behavi#ral adj2 practice).tw. | 150 |
| 11 | (behavi#ral adj2 rehearsal).tw. | 51 |
| 12 | mental rehearsal.tw. | 124 |
| 13 | (monitoring adj2 behavi?r*).tw. | 927 |
| 14 | reframing.tw. | 1292 |
| 15 | graded tasks.tw. | 15 |
| 16 | role model.tw. | 1258 |
| 17 | (reward adj2 behavi?r*).tw. | \| 1182 \|  \| \| --- \| --- \| |
| 18 | overcorrection.tw. | 1588 |
| 19 | problem solving.tw. | 15799 |
| 20 | ((prompt* or cue) adj2 (treatment or guideline)).tw. | 5865 |
| 21 | (re-attribution or reattribution).tw. | 70 |
| 22 | (restructur* adj2 environment).tw. | 26 |
| 23 | (review adj2 behavi?r adj2 (goal or goals)).tw. | 4 |
| 24 | (salience adj2 consequences).tw. | 7 |
| 25 | peer comparison.tw. | 88 |
| 26 | (shaping adj2 behavi?r*).tw. | 325 |
| 27 | (reinforcement or reinforcing or reinforcer).tw. | 44205 |
| 28 | (commitment adj2 (guideline or protocol)).tw. | 13 |
| 29 | (behavi?ral adj2 consequences).tw. | 1828 |
| 30 | (generali#ation adj2 behavi?r*).tw. | 103 |
| 31 | classical conditioning.tw. | 2450 |
| 32 | operant conditioning.tw. | 1908 |
| 33 | covert learning.tw. | 5 |
| 34 | shaping knowledge.tw. | 9 |
| 35 | (reattribution or re-attribution).tw. | 70 |
| 36 | habit reversal.tw. | 209 |
| 37 | habit formation.tw. | 336 |
| 38 | (rais* adj2 awareness).tw. | 8324 |
| 39 | external change agent.tw. | 6 |
| 40 | (guidance adj2 (manager or supervisor or "change leader" or champion or "implementation leader")).tw. | 3 |
| 41 | performance evaluation.tw. | 4291 |
| 42 | change leader.tw. | 14 |
| 43 | (knowledge adj2 transfer).tw. | 1895 |
| 44 | (computerized adj2 decisional adj2 support).tw. | 3 |
| 45 | (multiprofessional adj2 collaboration).tw. | 40 |
| 46 | or/1-45 | 122332 |
| 47 | (depress* or dysthymia or mood dysregulation or premenstrual dysphoric).tw. or depressive disorder/ | 419149 |
| 48 | (clinician? or practitioner? or pharmacist? or provider? or physician? or doctor? or counselor? or therapist? or psycholog* or psychiatr* or patient care team? or practice team?).tw. or (managed care or general practice).tw. | 1243672 |
| 49 | ((evidence based or guideline* or “care protocol” or treatment) adj2 (recommendation or recommended) adj2 (treatment or “appropriate care”)).tw. | 8930 |
| 50 | 46 and 47 and 48 and 49 | 1 |
| 51 | 47 and 48 and 49 | 164 |
| 52 | goal setting.tw. | 2958 |
| 53 | 47 and 48 and 49 and 52 | 0 |
| 54 | 48 or 49 | 1250945 |
| 55 | 46 and 47 and 54 | 1414 |
| 56 | Randomized controlled trial.pt. or (random$.tw. and (publisher or pubmed-not-medline or in process).st.) | 587329 |
| 57 | 55 and 56 | 272 |
| 58 | (mouse or mice or rats or dogs).ti. | 881577 |
| 59 | 57 not 58 | 271 |
| 60 | humans/ or (publisher or pubmed-not-medline or in process).st. | 20023064 |
| 61 | 59 and 60 | 270 |
| 62 | limit 61 to english language | 266 |
| 63 | limit 62 to yr="2017" | 14 |

PsycINFO
*Initial on 2/20/2017 and update on 8/28/2017*

**Human, English**

Depress* OR dysthymia OR “mood dysregulation” OR “premenstrual dysphoric” OR (DE "Major Depression" OR DE "Anaclitic Depression" OR DE "Dysthymic Disorder" OR DE "Endogenous Depression" OR DE "Late Life Depression" OR DE "Postpartum Depression" OR DE "Reactive Depression" OR DE "Recurrent Depression" OR DE "Treatment Resistant Depression")

AND

“knowledge translation” OR “knowledge transfer” OR “continuing education” OR “behavior intervention” OR “information campaign” OR “provider education” OR “opinion leader” OR “opinion champion” OR “academic detailing” OR “Train the trainer” OR “depression education” OR “continuing education” OR “competence training” OR “learning collaborative” OR “guideline adherence” OR “guideline rollout” OR “guideline roll-out” OR “guideline toolkit” OR “provider training” OR “provider reminder” OR reminder* OR “clinical support tool” OR “guideline prompt” OR “guideline prompting” OR “guideline cue” OR “behavior feedback” OR “patient care planning” OR “computer assisted drug therapy” OR “continuous quality improvement” OR CQI OR TQM OR “total quality management” OR “process improvement” OR “model for improvement” OR PDSA OR PDCA OR TQIS OR “Plan do study” OR “Plan do check” OR “shewart cycle” OR “shewhart cycle” OR “deming cycle” OR “shewart method” OR “shewhart method” OR “deming method” OR “breakthrough series” OR “collaborative breakthrough” OR “breakthrough collaborative” OR “six sigma” OR persuasion OR “persuasive communication” OR incentivize OR incentivise OR incentivisation OR incentivization OR incentive OR “behavioural modeling” OR “behavioral modeling” OR “provider behavior” OR “provider behaviour” OR “behavior substitution” OR “behaviour substitution” OR “behavior contract” OR “behaviour contract” OR “cue signaling” OR “mental rehearsal” OR “behavior monitoring” OR “behaviour monitoring” OR reframing OR “role model” OR “behavior reward” OR “behaviour reward” OR “behavior overcorrection” OR “behaviour overcorrection” OR “shaping behavior” OR “shaping behaviour” OR reinforcement OR reinforcing OR reinforce OR “guideline commitment” OR “covert learning” OR “shaping knowledge” OR “habit reversal” OR “habit formation” OR “raising awareness” OR “raise awareness” OR “external change agent” OR “performance evaluation” OR “change leader” OR “computerized decision support” OR “goal setting”

AND

Clinician* OR practitioner* OR pharmacist* OR provider* OR physician* OR doctor* OR counselor* OR therapist* OR psycholog* OR psychiatr* OR “patient care team” OR “patient care teams” OR “managed care” OR “general practice”

AND

(“evidence based” OR “evidence-based” OR guideline* OR “care protocol” OR “treatment recommendation*” OR “recommended treatment*”)

AND

DE clinical trials OR random*

**Results: 99**

CINAHL
*Initial on 2/20/2017 and update on 8/28/2017*

(MH "Depression+") OR (MH "Premenstrual Dysphoric Disorder") OR depress* OR dysthymia OR "mood dysregulation" OR "premenstrual dysphoric"

AND

“knowledge translation” OR “knowledge transfer” OR “continuing education” OR “behavior intervention” OR “information campaign” OR “provider education” OR “opinion leader” OR “opinion champion” OR “academic detailing” OR “Train the trainer” OR “depression education” OR “continuing education” OR “competence training” OR “learning collaborative” OR “guideline adherence” OR “guideline rollout” OR “guideline roll-out” OR “guideline toolkit” OR “provider training” OR “provider reminder” OR reminder* OR “clinical support tool” OR “guideline prompt” OR “guideline prompting” OR “guideline cue” OR “behavior feedback” OR “patient care planning” OR “computer assisted drug therapy” OR “continuous quality improvement” OR CQI OR TQM OR “total quality management” OR “process improvement” OR “model for improvement” OR PDSA OR PDCA OR TQIS OR “Plan do study” OR “Plan do check” OR “shewart cycle” OR “shewhart cycle” OR “deming cycle” OR “shewart method” OR “shewhart method” OR “deming method” OR “breakthrough series” OR “collaborative breakthrough” OR “breakthrough collaborative” OR “six sigma” OR persuasion OR “persuasive communication” OR incentivize OR incentivise OR incentivisation OR incentivization OR incentive OR “behavioural modeling” OR “behavioral modeling” OR “provider behavior” OR “provider behaviour” OR “behavior substitution” OR “behaviour substitution” OR “behavior contract” OR “behaviour contract” OR “cue signaling” OR “mental rehearsal” OR “behavior monitoring” OR “behaviour monitoring” OR reframing OR “role model” OR “behavior reward” OR “behaviour reward” OR “behavior overcorrection” OR “behaviour overcorrection” OR “shaping behavior” OR “shaping behaviour” OR reinforcement OR reinforcing OR reinforce OR “guideline commitment” OR “covert learning” OR “shaping knowledge” OR “habit reversal” OR “habit formation” OR “raising awareness” OR “raise awareness” OR “external change agent” OR “performance evaluation” OR “change leader” OR “computerized decision support” OR “goal setting”

AND

Clinician* OR practitioner* OR pharmacist* OR provider* OR physician* OR doctor* OR counselor* OR therapist* OR psycholog* OR psychiatr* OR “patient care team” OR “patient care teams” OR “managed care” OR “general practice”

AND

(“evidence based” OR “evidence-based” OR guideline* OR “care protocol” OR “treatment recommendation*” OR “recommended treatment*”)

AND

(MH "Randomized Controlled Trials") OR random*

**Results: 32 – duplicates = 17**

CENTRAL

*Initial on 2/20/17 and update on 8/28/17*

**(title/abs/key)
2017-2017**

depress* OR dysthymia OR "mood dysregulation" OR "premenstrual dysphoric"

AND

“knowledge translation” OR “knowledge transfer” OR “continuing education” OR “behavior intervention” OR “information campaign” OR “provider education” OR “opinion leader” OR “opinion champion” OR “academic detailing” OR “Train the trainer” OR “depression education” OR “continuing education” OR “competence training” OR “learning collaborative” OR “guideline adherence” OR “guideline rollout” OR “guideline roll-out” OR “guideline toolkit” OR “provider training” OR “provider reminder” OR reminder* OR “clinical support tool” OR “guideline prompt” OR “guideline prompting” OR “guideline cue” OR “behavior feedback” OR “patient care planning” OR “computer assisted drug therapy” OR “continuous quality improvement” OR CQI OR TQM OR “total quality management” OR “process improvement” OR “model for improvement” OR PDSA OR PDCA OR TQIS OR “Plan do study” OR “Plan do check” OR “shewart cycle” OR “shewhart cycle” OR “deming cycle” OR “shewart method” OR “shewhart method” OR “deming method” OR “breakthrough series” OR “collaborative breakthrough” OR “breakthrough collaborative” OR “six sigma” OR persuasion OR “persuasive communication” OR incentivize OR incentivise OR incentivisation OR incentivization OR incentive OR “behavioural modeling” OR “behavioral modeling” OR “provider behavior” OR “provider behaviour” OR “behavior substitution” OR “behaviour substitution” OR “behavior contract” OR “behaviour contract” OR “cue signaling” OR “mental rehearsal” OR “behavior monitoring” OR “behaviour monitoring” OR reframing OR “role model” OR “behavior reward” OR “behaviour reward” OR “behavior overcorrection” OR “behaviour overcorrection” OR “shaping behavior” OR “shaping behaviour” OR reinforcement OR reinforcing OR reinforce OR “guideline commitment” OR “covert learning” OR “shaping knowledge” OR “habit reversal” OR “habit formation” OR “raising awareness” OR “raise awareness” OR “external change agent” OR “performance evaluation” OR “change leader” OR “computerized decision support” OR “goal setting”

AND

Clinician* OR practitioner* OR pharmacist* OR provider* OR physician* OR doctor* OR counselor* OR therapist* OR psycholog* OR psychiatr* OR “patient care team” OR “patient care teams” OR “managed care” OR “general practice”

AND

(“evidence based” OR “evidence-based” OR guideline* OR “care protocol” OR “treatment recommendation*” OR “recommended treatment*”)

(adding AND random* removes 5 so I just left the in, since this is a databases of controlled trials)

**Results: 112- duplicates = 67**

CDSR

*Initial on 2/20/17 and update on 8/28/17*

**(title/abstract/keywords)
2017-2017**

depress* OR dysthymia OR "mood dysregulation" OR "premenstrual dysphoric"

AND

“knowledge translation” OR “knowledge transfer” OR “continuing education” OR “behavior intervention” OR “information campaign” OR “provider education” OR “opinion leader” OR “opinion champion” OR “academic detailing” OR “Train the trainer” OR “depression education” OR “continuing education” OR “competence training” OR “learning collaborative” OR “guideline adherence” OR “guideline rollout” OR “guideline roll-out” OR “guideline toolkit” OR “provider training” OR “provider reminder” OR reminder* OR “clinical support tool” OR “guideline prompt” OR “guideline prompting” OR “guideline cue” OR “behavior feedback” OR “patient care planning” OR “computer assisted drug therapy” OR “continuous quality improvement” OR CQI OR TQM OR “total quality management” OR “process improvement” OR “model for improvement” OR PDSA OR PDCA OR TQIS OR “Plan do study” OR “Plan do check” OR “shewart cycle” OR “shewhart cycle” OR “deming cycle” OR “shewart method” OR “shewhart method” OR “deming method” OR “breakthrough series” OR “collaborative breakthrough” OR “breakthrough collaborative” OR “six sigma” OR persuasion OR “persuasive communication” OR incentivize OR incentivise OR incentivisation OR incentivization OR incentive OR “behavioural modeling” OR “behavioral modeling” OR “provider behavior” OR “provider behaviour” OR “behavior substitution” OR “behaviour substitution” OR “behavior contract” OR “behaviour contract” OR “cue signaling” OR “mental rehearsal” OR “behavior monitoring” OR “behaviour monitoring” OR reframing OR “role model” OR “behavior reward” OR “behaviour reward” OR “behavior overcorrection” OR “behaviour overcorrection” OR “shaping behavior” OR “shaping behaviour” OR reinforcement OR reinforcing OR reinforce OR “guideline commitment” OR “covert learning” OR “shaping knowledge” OR “habit reversal” OR “habit formation” OR “raising awareness” OR “raise awareness” OR “external change agent” OR “performance evaluation” OR “change leader” OR “computerized decision support” OR “goal setting”

AND

Clinician* OR practitioner* OR pharmacist* OR provider* OR physician* OR doctor* OR counselor* OR therapist* OR psycholog* OR psychiatr* OR “patient care team” OR “patient care teams” OR “managed care” OR “general practice”

AND

(“evidence based” OR “evidence-based” OR guideline* OR “care protocol” OR “treatment recommendation*” OR “recommended treatment*”)

**Results= 2 (no dups)**

**Appendix B:** *Critical Appraisal Ratings Using the Cochrane Risk of Bias tool and the QI-MQCS*

| **Study ID** | **Random Sequence Generation (selection bias)** | **Allocation Concealment (selection bias)** | **Blinding of Participants and Providers (performance bias)** | **Blinding of Outcome Assessors (detection bias)** | **Completeness of Reporting Outcome Data (attrition bias)** | **Selective Outcome Reporting (reporting bias)** | **Cross-overs/ contamination (contamination bias)** | **1. Organizational Motivation**  *Organizational problem/reason or motivation for intervention* | **2. Intervention Rationale**  *Rationale linking the intervention to expected effects* | **3. Intervention**  *Specific changes in healthcare delivery organization/structure* | **4. Organizational Characteristics**  *Organizational demographics and basic characteristics* | **5. Implementation**  *Approach to designing and/or introducing organizational changes* | **6. Study Design**  *Study design and comparator* | **7. Comparator**  *Information about comparator care processes* | **8. Data Source**  *Data sources and outcome definition* | **9. Timing**  *Timing of intervention and evaluation* | **10. Adherence/Fidelity**  *Adherence to the intervention* | **11. Health Outcomes**  *Patient health-related outcome* | **12. Organizational Readiness**  *Organizational readiness for the studied intervention* | **13. Penetration/\Reach**  *Penetration/reach of the intervention* | **14. Sustainability**  *Potential for intervention maintenance or sustainability* | **15. Spread**  *Ability to be spread or replicated* | **16. Limitations**  *Quality of the interpretation of findings* | **Summary Rating** |
| --- | --- | --- | --- | --- | --- | --- | --- | --- | --- | --- | --- | --- | --- | --- | --- | --- | --- | --- | --- | --- | --- | --- | --- | --- |
| Aakhus, 2016 | Low | U | High | Low | Low | Low | U | Met | Met | Met | Met | Met | Met | Not met | Met | Not met | Not met | Met | Met | Met | Not met | Not met | Met | Good |
| Azocar, 2003 | U | U | High | U | U | U | U | Met | Met | Not met | Met | Met | Met | Not met | Met | Not met | Met | Not met | Met | Met | Not met | Not met | Met | Poor |
| Baker, 2001 | Low | U | High | Low | U | U | Low | Met | Met | Met | Met | Met | Met | Met | Met | Not met | Met | Met | Met | Met | Met | Met | Met | Good |
| Bosmans, 2006 | U | U | High | Low | Low | Low | Low | Met | Met | Met | Met | Met | Met | Met | Met | Not met | Met | Not met | Not Met | Not met | Not met | Not met | Met | Good |
| Callahan, 1994 | U | U | High | U | U | U | Low | Met | Met | Met | Met | Met | Met | Met | Met | Not met | Met | Met | Not Met | Met | Not met | Not met | Met | Poor |
| Datto, 2003 | U | U | High | U | U | U | Low | Met | Met | Met | Met | Met | Met | Met | Met | Not met | Met | Met | Met | Met | Not met | Not met | Met | Poor |
| Eccles, 2007 | Low | U | High | Low | High | U | Low | Met | Met | Met | Met | Met | Met | Not met | Met | Met | Not met | Not met | Met | Not met | Not met | Not met | Met | Fair |
| Freemantle, 2002 | Low | U | High | U | U | Low | Low | Met | Met | Met | Met | Met | Met | Met | Not met | Not met | Not met | Not met | Met | Met | Not met | Not met | Met | Fair |
| Gerrity, 1999 | U | U | High | Low | Low | U | U | Met | Met | Met | Not met | Met | Met | Not met | Met | Met | Met | Not met | Not Met | Not met | Not met | Not met | Met | Fair |
| Goldberg, 1998 | U | U | High | U | High | Low | Low | Met | Met | Met | Met | Met | Met | Met | Met | Met | Met | Met | Met | Met | Not met | Not met | Met | Fair |
| Keeley, 2014 | Low | U | High | U | Low | U | Low | Met | Met | Met | Met | Met | Met | Met | Met | Not met | Not met | Met | Not Met | Met | Not met | Not met | Met | Good |
| Kurian, 2009 | U | U | High | Low | U | U | U | Met | Met | Met | Met | Met | Met | Met | Met | Met | Met | Met | Met | Not met | Not met | Not met | Met | Fair |
| Lin, 2001 | U | U | High | U | U | U | High | Met | Met | Met | Met | Met | Met | Met | Met | Met | Met | Not met | Not Met | Met | Not met | Not met | Met | Poor |
| Linden, 2008 | U | U | High | U | U | U | U | Met | Met | Met | Met | Met | Met | Met | Met | Not met | Not met | Met | Not Met | Not met | Not met | Not met | Met | Poor |
| Nilsson, 2001 | U | U | High | U | High | U | U | Met | Met | Met | Met | Met | Met | Met | Met | Not met | Met | Not met | Not Met | Met | Not met | Met | Met | Poor |
| Rollman, 2001 | Low | U | High | Low | Low | U | High | Met | Met | Met | Met | Met | Met | Met | Met | Not met | Met | Met | Met | Not met | Not met | Met | Met | Good |
| Shirazi, 2013 | U | U | High | U | Low | U | Low | Met | Met | Met | Met | Met | Met | Met | Met | Not met | Met | Not met | Not Met | Met | Not met | Not met | Met | Fair |
| Simon, 2000 | Low | U | High | Low | U | U | High | Met | Met | Met | Met | Met | Met | Met | Met | Met | Not met | Met | Not Met | Not met | Not met | Not met | Met | Fair |
| Sinnema, 2015 | Low | Low | High | U | Low | U | Low | Met | Met | Met | Met | Met | Met | Met | Met | Met | Met | Met | Met | Met | Not met | Not met | Met | Good |
| van Eijk, 2001 | U | U | High | U | Low | U | Low | Met | Met | Met | Not met | Met | Met | Not met | Met | Met | Not met | Not met | Not Met | Not met | Not met | Not met | Met | Fair |
| Worrall, 1999 | Low | U | High | High | U | U | Low | Met | Met | Met | Met | Met | Met | Met | Not met | Not met | Not met | Met | Not Met | Not met | Not met | Not met | Met | Fair |
| Yawn, 2012 | U | U | High | Low | U | U | Low | Met | Met | Not met | Met | Met | Met | Met | Met | Met | Met | Met | Met | Not met | Not met | Not met | Met | Fair |

Note: U = unclear risk of bias, Low = low risk of bias, High = high risk of bias. All studies were de facto considered high risk for personnel blinding.

**Appendix C** *Detailed Quality of Evidence and Summary of Findings*

| **Intervention type and outcome measure** | **Number of RCTs and Participants** | **Study Limitations** | **Inconsistency** | **Indirectness** | **Imprecision** | **Publication bias** | **Control Risk/Score** | **Intervention Risk/Score** | **Direction and**  **Magnitude of Relative Effect** | **Absolute control Risk / Score** | **Absolute Risk/Score Difference** | **GRADE** |
| --- | --- | --- | --- | --- | --- | --- | --- | --- | --- | --- | --- | --- |
| **KQ1. Effects of provider intervention on healthcare professional behavior** |  |  |  |  |  |  |  |  |  |  |  |  |
| **Provider intervention vs UCP** |  |  |  |  |  |  |  |  |  |  |  |  |
| Odds of achieved provider adherence (main indication) | 13 RCTs [51, 52, 57, 58, 60-62, 64, 78-82]  N = 3,158 | -- | ^ (H) | D | (P) | NC | N/A | 741/1567 | Provider interventions not statistically significantly different from comparator groups (OR 1.60; CI 0.76, 3.37) | N/A* | n.s. | Moderate |
| Mean difference in achieved provider adherence (main indication) | 9 RCTs [52, 60, 62, 63, 83-87]  N = 1,236 | -- | ^^ (H) (D) | D | (P) | NC | N/A | N/A | Provider interventions not statistically significantly different from comparator groups (SMD 0.17; CI -0.16, 0.50) | N/A* | -- | Low |
| Incidence rate of achieved provider adherence (main indication) | 4 RCTs [51, 65, 78, 87]  N = 63,588 | -- | ^ (H) | D | ^ | NC | N/A | N/A | Provider interventions not statistically significantly different from comparator groups (IRR 1.16; CI 0.63, 2.14) | N/A* | -- | Low |
| Odds of improved medication prescribing | 11 RCTs [51, 52, 57, 58, 60-62, 64, 78, 80, 81]  N = 4,116 | -- | ^ (H) | D | ^ | n.s. | 788/2078 | 915/2038 | Provider interventions statistically significantly different from comparator groups (OR 1.42; CI 1.04, 1.92) favoring the intervention | 390/1000 | 55 more per 1000 | Low |
| Mean difference in improved medication prescribing | 3 RCTs [85-87]  N = 414 | -- | ^ (D) | D | ^ | N/A | N/A | N/A | Provider interventions not statistically significantly different from comparator groups (SMD 0.15; CI -0.48, 0.79) | N/A* | -- | Low |
| Incidence rate of improved medication prescribing | 3 RCTs [65, 78, 87]  N= 63,144 | -- | ^ (H) | D | ^ | N/A | N/A | N/A | Provider interventions not statistically significantly different from comparator group (IRR 1.02; CI 0.44, 2.36) | N/A* | -- | Low |
| Odds for increased contact with patients | 3 RCTs [61, 64, 81]  N = 710 | -- | ^ (H) | D | ^ | N/A | 44/345 | 134/365 | Provider interventions not statistically significantly different from comparator groups (OR 6.40; CI 0.13, 322.40) | 360/1000 | n.s. | Low |
| Mean difference in contact with patients | 3 RCTs [52, 60, 62]  N = 225 | -- | -- | D | ^ | N/A | N/A | N/A | Provider interventions not statistically significantly different from comparator groups (SMD 0.17; CI -0.84, 1.19) | N/A* | -- | Moderate |
| Incidence rate of number of consultations (contact with patients) | 1 RCT [51]  N=444 | --- | ^^ (S) | D | (P) | NC | N/A | N/A | Provider intervention statistically significantly different from comparator group (IRR 1.78; CI 1.14, 2.78) favoring the intervention | N/A* | -- | Very low |
| Odds of general adherence to intervention | 6 RCTs [57, 61, 64, 79, 81, 82]  N=1,375 | -- | ^ (H) | D | ^ | N/A | 374/676 | 479/699 | Provider interventions not statistically significantly different from comparator groups (OR 2.26; CI 0.50, 10.28) | 465/1000 | n.s. | Low |
| Mean difference in general adherence to intervention | 3 RCTs [63, 83, 84]  N =597 | -- | ^^ (H) (D) | D | ^ | N/A | N/A | N/A | Provider interventions not statistically significantly different from comparator groups (SMD 0.23; CI -1.42, 1.89) | N/A* | -- | Very low |
| Odds of referral offered to patient | 4 RCTs [51, 61, 62, 80]  N = 896 |  | -- | D | ^ | N/A | 44/439 | 54/457 | Provider interventions not statistically significantly different from comparator groups (OR 1.11; CI 0.33, 3.70) | 93/1000 | n.s. | Moderate |
| **Provider intervention vs practice redesign** |  |  |  |  |  |  |  |  |  |  |  |  |
| Odds of achieved provider adherence (main indication) | 3 RCTs [52, 53, 58]  N = 867 | -- | -- | D | ^ | N/A | N/A | N/A | Provider interventions not statistically significantly different from comparator groups (OR 0.81; CI 0.30, 2.19) | N/A | n.s. | Moderate |
| Mean difference in achieved provider adherence (main indication) | 1 RCT [52]  N = 24 | -- | ^^ (S) | D | (P) | N/A | 0.09 | 0.13 | Provider intervention not statistically significantly different from comparator group (SMD 0.07; CI -0.73, 0.87) | N/A | 0.04 | Low |
| Odds of improved medication prescribing | 2 RCTs [52, 58]  N = 1,738 | -- | ^ (D) | D | ^ | N/A | 275/853 | 294/885 | Provider interventions not statistically significantly different from comparator groups (OR 0.96; CI 0.18, 5.08) | 375/1000 | n.s. | Low |
| Mean difference in contact with patients | 1 RCT [52]  N = 24 | -- | ^^ (S) | D | (P) | N/A | 0.09 | 0.13 | Provider intervention not statistically significantly different from comparator group (SMD 0.07; CI -0.73, 0.87) | N/A | 0.04 | Low |
| Odds of general adherence to intervention | 1 RCT [53]  N = 61 | Poor RoB, IP | ^^ (S) | D | (P) | N/A | N/A | N/A | Provider interventions not statistically significantly different from comparator groups (OR 0.30; CI 0.08, 1.14) | N/A | n.s. | Very low |
| **Provider intervention vs other interventions** |  |  |  |  |  |  |  |  |  |  |  |  |
| Odds of achieved provider adherence (main indication) | 1 RCT: [59]  N = 171 | PND | ^^ (S) | D | ^ | N/A | 36/85 | 33/86 | Provider intervention not statistically significantly different from comparator group (OR 0.85; CI 0.43, 1.69) | 420/1000 | n.s. | Very low |
| Odds of improved medication prescribing | 1 RCT [59]  N = 171 | PND | ^^ (S) | D | ^ | N/A | 36/85 | 33/86 | Provider intervention not statistically significantly different from comparator group (OR 0.85; CI 0.43, 1.69) | 420/1000 | n.s. | Very low |
| Odds of general adherence to intervention | 1 RCT [59]  N = 171 | PND | ^^ (S) | D | ^ | N/A | 29/85 | 16/86 | Provider intervention not statistically significantly different from comparator group (OR 0.45; C 0.20, 1.01) | 340/1000 | n.s. | Very low |
| **KQ1a. Effects by intervention type** |  |  |  |  |  |  |  |  |  |  |  |  |
| **Comparative effectiveness** |  |  |  |  |  |  |  |  |  |  |  |  |
| Guideline distribution plus implementation recommendations vs guideline distribution alone:  Odds of achieved provider adherence (main indication) | 1 RCT [57]  N = 378 | IP | ^^ (S) | D | ^ | N/A | 168/181 | 188/197 | Provider interventions not statistically significantly different (OR 1.62; CI 0.64, 4.06) | 928/1000 | n.s. | Very low |
| Guideline distribution and education vs guideline distribution, education, and nurse disease management (system redesign): Odds of achieved provider adherence (main indication) | 1 RCT [53]  N = 61 | Poor RoB, IP | ^^ (S) | D | ^ | N/A | N/A | N/A | Provider interventions not statistically significantly different (OR 0.30; CI 0.08, 1.14) | N/A | n.s. | Very low |
| Academic detailing vs academic detailing plus continuous quality improvement: Odds of achieved provider adherence (main indication) | 1 RCT [58]  N = 389 | -- | ^^ (S) | D | ^ | N/A | 36/240 | 22/149 | Provider interventions not statistically significantly different (OR 1.01; CI 0.48, 2.11) | 148/1000 | n.s. | Very low |
| Guideline distribution vs guideline distribution and motivational interviewing training:  Odds for achieved provider adherence (main indication) | 1 RCT [59]  N = 171 | PND | ^^ (S) | D | ^ | N/A | 36/85 | 33/86 | Provider interventions not statistically significantly different (OR 0.85; CI 0.43, 1.69) | 420/1000 | n.s. | Very low |
| Education plus additional training sessions vs education alone:  Odds for achieved provider adherence (main indication) | 1 RCT [60]  N = 55 | PND | ^^ (S) | D | ^ | N/A | 15/23 | 22/32 | Provider interventions not statistically significantly different (OR 1.17; CI 0.33, 4.19) | 652/1000 | n.s. | Very low |
| Education plus additional training sessions vs education alone:  Mean difference in achieved provider adherence (main indication) | 1 RCT [60]  N = 55 | PND | ^^ (S) | D | ^ | N/A | 3.70 | 5.00 | Provider interventions not statistically significantly different (SMD 0.67; CI 0.06, 1.28) | N/A | 1.30 | Very low |
| Patient-specific treatment recommendations vs recommendations and care management:  Odds for achieved provider adherence (main indication) | 1 RCT [52]  N = 417 | -- | ^^ (S) | D | ^ | N/A | 92/196 | 95/221 | Provider interventions not statistically significantly different (OR 0.85; CI 0.58, 1.25) | 470/1000 | n.s. | Very low |
| Patient-specific treatment recommendations vs recommendations and care management:  Mean difference in achieved provider adherence (main indication) | 1 RCT [52]  N = 417 | -- | ^^ (S) | D | ^ | N/A | 0.09 | 0.13 | Provider interventions not statistically significantly different (SMD 0.07; CI -0.73, 0.87). | N/A | 0.04 | Very low |
| Training plus tailored implementation vs training alone:  Odds for achieved provider adherence (main indication) | 1 RCT [51]  N = 444 | -- | ^^ (S) | D | ^ | N/A | 30/246 | 26/198 | Provider interventions not statistically significantly different (OR 1.07; CI 0.52, 2.19). | 122/1000 | n.s. | Very low |
| Training plus tailored implementation vs training alone:  Incidence rate for achieved provider adherence (main indication) | 1 RCT [51]  N = 444 | -- | ^^ (S) | D | ^ | NC | N/A | N/A | Provider interventions statistically significantly different (IRR 1.78; CI 1.14, 2.78), favoring the intervention of training plus tailored implementation | N/A | -- | Very low |
| Guideline distribution plus workshop and consultation vs guideline distribution alone:  Odds of achieved provider adherence (main indication) | 1 RCT [62]  N = 147 | -- | ^^ (S) | D | ^ | N/A | 50/56 | 83/91 | Provider interventions not statistically significantly different (OR 1.25; CI 0.40, 3.90) | 893/1000 | n.s. | Very low |
| Guideline distribution plus workshop and consultation vs guideline distribution alone:  Mean difference in achieved provider adherence (main indication) | 1 RCT [62]  N = 147 | -- | ^^ (S) | D | ^ | N/A | 4.20 | 3.60 | Provider interventions not statistically significantly different (SMD -0.08; CI -0.42, 0.26) | N/A | -0.60 | Very low |
| Education plus other components vs guidelines and education without tailoring to stages of change:  Mean difference in achieved provider adherence (main indication) | 1 RCT [63]  N = 36 | -- | ^^ (S) | D | ^ | NC | 22.00 | 49.00 | Provider interventions statistically significantly different (SMD 0.89; CI 0.59, 1.18), favoring intervention with education plus other components tailored toward stages to change | N/A | 27.00 | Very low |
| Guideline distribution (passive) vs guideline distribution (active):  Odds of achieved provider adherence (main indication) | 1 RCT [61]  N = 138 | IP | ^^ (S) | D | ^ | N/A | 54/68 | 61/70 | Provider interventions not statistically significantly different (OR 1.76; CI 0.64, 4.86) | 794/1000 | n.s. | Very low |
| **Indirect comparison** |  |  |  |  |  |  |  |  |  |  |  |  |
| Meta-regression education only vs education plus for odds of achieved provider adherence (main indication) | 10 RCTs [51, 52, 58, 60, 62, 64, 78, 79, 81, 82]  N = 2,957 | N/A | N/A | ^^ (I) | ^ | N/A | N/A | N/A | No systematic effect detected (p = 0.574) | N/A | N/A | Very low |
| Meta-regression education only vs education plus for mean difference in achieved provider adherence (main indication) | 8 RCTs [52, 60, 62, 63, 83, 85-87]  N = 712 | N/A | N/A | ^^ (I) | ^ | N/A | N/A | N/A | No systematic effect detected (p = 0.238) | N/A | N/A | Very low |
| Meta-regression unidimensional vs multidimensional for odds of achieved provider adherence (main indication) | 13 RCTs [52, 57, 58, 60-63, 78-82, 86]  N = 2,953 | N/A | N/A | ^^ (I) | ^ | N/A | N/A | N/A | No systematic effect detected (p = 0.707) | N/A | N/A | Very low |
| Meta-regression unidimensional vs multidimensional for mean difference in achieved provider adherence (main indication) | 9 RCTs [52, 60, 62, 63, 83-87]  N = 1,236 | N/A | N/A | ^^ (I) | ^ | N/A | N/A | N/A | No systematic effect detected (p = 0.055) | N/A | N/A | Very low |
| Meta-regression unidimensional vs multidimensional for odds of improved medical prescribing | 12 RCTs [51, 52, 57-62, 64, 78, 80, 81]  N = 2,678 | N/A | N/A | ^^ (I) | ^ | N/A | N/A | N/A | No systematic effect detected (p = 0.317) | N/A | N/A | Very low |
| Meta-regression unidimensional vs multidimensional for odds of referral offered to patients | 4 RCTs [51, 61, 62, 80]  N = 896 | N/A | N/A | ^^ (I) | ^ | N/A | N/A | N/A | No systematic effect detected (p = 0.195) | N/A | N/A | Very low |
| Meta-regression intervention intensity for odds of achieved provider adherence (main indication) | 13 RCTs [51, 52, 57, 58, 60-62, 64, 78-82]  N = 3,158 | N/A | N/A | ^^ (I) | ^ | N/A | N/A | N/A | No systematic effect detected (p = 0.973) | N/A | N/A | Very low |
| Meta-regression intervention intensity for mean difference in achieved provider adherence (main indication) | 9 RCTs [52, 60, 62, 63, 83-87]  N = 1,236 | N/A | N/A | ^^ (I) | ^ | NC | N/A | N/A | The analysis suggested that the intensity of the intervention is associated with the effect size (p = 0.033) | N/A | N/A | Very low |
| Meta-regression intervention intensity for odds of improved medical prescribing | 12 RCTs [51, 52, 57-62, 64, 78, 80, 81]  N = 2,678 | N/A | N/A | ^^ (I) | ^ | N/A | N/A | N/A | No systematic effect detected (p = 0.414) | N/A | N/A | Very low |
| Meta-regression intervention intensity for odds of general adherence to intervention | 8 RCTs [53, 57, 59, 61, 64, 79, 81, 82]  N = 2,411 | N/A | N/A | ^^ (I) | ^ |  | NA | NA | No systematic effect detected (p = 0.542) | NA | NA | Very low |
| **Subgroup analyses**  **by intervention type** |  |  |  |  |  |  |  |  |  |  |  |  |
| Guideline distribution only:  Odds of achieved provider adherence (main indication) | 3 RCTs [57, 61, 80]  N = 683 | N/A | -- | D | ^ | N/A | N/A | N/A | Provider interventions not statistically significantly different from comparator groups (OR 1.28; CI 0.75, 2.19) | N/A | N/A | Low |
| Guideline distribution only:  Mean difference for achieved provider adherence (main indication) | 1 RCT [84]  N = 281 | PND | ^^ (S) | D | ^ | NC | 0.91 | 0.80 | Provider intervention statistically significantly different from comparator group (SMD -0.44; CI -0.68, -0.20), favoring the comparator | N/A | -0.11 | Very low |
| Guideline distribution only:  Odds of improved medication prescribing | 4 RCTs [57, 59, 61, 80]  N = 854 | N/A | ^ (H) | D | ^ | N/A | N/A | N/A | Provider interventions not statistically significantly different from comparator groups (OR 1.52; CI 0.60, 3.86) | N/A | N/A | Low |
| Guideline distribution only:  Odds of increased provider contact with patients | 1 RCT [61]  N = 130 | IP | ^^ (S) | D | ^ | N/A | 26/62 | 45/68 | Provider intervention statistically significantly different from comparator group (OR 2.71; CI 1.24, 5.94) | 419/1000 | 242 more per 1000 | Very low |
| Guideline distribution only:  Odds of general adherence to intervention | 3 RCTs [57, 59, 61]  N = 679 | N/A | ^ (H) | D | ^^ | N/A | N/A | N/A | Provider interventions not statistically significantly different from comparator groups (OR 0.95; CI 0.17, 5.17) | N/A | N/A | Very low |
| Education only:  Odds of achieved provider adherence (main indication) | 3 RCTs [79, 81, 82]  N = 338 | N/A | ^ (H) | D | ^ | N/A | N/A | N/A | Provider interventions not statistically significantly different from comparator groups (OR 3.04; CI 0.01, 756.17) | N/A | N/A | Low |
| Education only:  Mean difference in achieved provider adherence (main indication) | 3 RCTs [85-87]  N = 414 | N/A | -- | D | ^ | N/A | N/A | N/A | Provider interventions not statistically significantly different from comparator groups (SMD 0.15; CI -0.48, 0.79) | N/A | N/A | Moderate |
| Education only:  Odds of improved medication prescribing | 1 RCT [81]  N = 48 | N/A | ^ (S) | D | ^ | N/A | N/A | N/A | Provider intervention not statistically significantly different from comparator group (OR 2.78; CI 0.80, 9.59) | N/A | N/A | Very low |
| Education only:  Odds of increased provider contact with patients | 1 RCT [81])  N = 48 | N/A | ^ (S) | D | ^ | N/A | 9/26 | 17/22 | Provider intervention statistically significantly different from comparator group (OR 6.42; CI 1.78, 23.18) | 346/1000 | 427 more per 1000 | Very low |
| Education only:  Odds of general adherence to intervention | 4 RCTs [53, 79, 81, 82]  N = 399 | N/A | ^ (H) | D | ^^ | N/A | N/A | N/A | Provider interventions not statistically significantly different from comparator groups (OR 2.03; CI 0.06, 73.30) | N/A | N/A | Very low |
| Education plus other components:  Odds for achieved provider adherence (main indication) | 7 RCTs [51, 52, 58, 60, 62, 64, 78]  N = 2090 | N/A | -- | D | ^ | N/A | N/A | N/A | Provider interventions not statistically significantly different from comparator groups (OR 1.17; CI 0.62, 2.18) | N/A | N/A | Moderate |
| Education plus other components:  Mean difference in achieved provider adherence (main indication) | 5 RCTs [52, 60, 62, 63, 83]  N =938 | N/A | ^ (H) | D | ^ | N/A | N/A | N/A | Provider interventions not statistically significantly different from comparator groups (SMD 0.37; CI -0.16, 0.90) | N/A | N/A | Low |
| Education plus other components:  Odds of improved medical prescribing | 7 RCTs [51, 52, 58, 60, 62, 64, 78]  N = 1710 | N/A | ^^ (H) | D | (P) | N/A | N/A | N/A | Provider interventions not statistically significantly different from comparator groups (OR 1.21; CI 0.85, 1.71) | N/A | N/A | Low |
| Education plus other components:  Odds of increased provider contact with patients | 1 RCT [64]  N = 483 | N/A | ^^ (S) | D | ^ | N/A | 0/233 | 55/250 | Provider interventions statistically significantly different from comparator group (OR 101.34; CI 6.17, 1664.08) | 0/1000 | 220 more per 1000 | Very low |
| Education plus other components:  Odds of general adherence to intervention | 1 RCT [64]  N = 482 | N/A | ^^ (S) | D | (P) | N/A | 70/189 | 176/293 | Provider interventions statistically significantly different from comparator group (OR 2.56; CI 1.65, 3.97) | 370/1000 | 230 more per 1000 | Very low |
| **KQ1b. Effects by provider type** |  |  |  |  |  |  |  |  |  |  |  |  |
| Meta-regression single provider vs team for odds of achieved provider adherence (main indication) | 13 RCTs [51, 52, 57, 58, 60-62, 64, 78-82]  N = 3,158 | N/A | N/A | ^^ (I) | ^ | NC | N/A | N/A | The analysis suggested that the type of provider is associated with the effect size (p = 0.034); however, the analysis is based on only 1 team intervention | N/A | N/A | Very low |
| **Subgroup analysis by provider type** |  |  |  |  |  |  |  |  |  |  |  |  |
| Single provider interventions:  Odds for achieved provider adherence (main indication) | 12 RCTs [51, 52, 57, 58, 60-62, 78-82]  N =1334 | N/A | ^ (H) | D | ^ | N/A | N/A | N/A | Provider interventions not statistically significantly different from comparator groups (OR 1.42; CI 0.74, 2.73) | N/A | N/A | Low |
| Team provider interventions:  Odds of achieved provider adherence (main indication) | 1 RCT [64]  N =482 | N/A | ^^ (S) | D | ^ | NC | N/A | N/A | Provider intervention statistically significantly different from comparator group (OR 101.34, CI 6.17, 1664.08), favoring the intervention | N/A | N/A | Very low |
| **KQ1c. Effect by setting** |  |  |  |  |  |  |  |  |  |  |  |  |
| Meta-regression primary care vs specialty care setting for mean difference in achieved adherence (main indication) | 9 RCTs [52, 60, 62, 63, 83-87]  N = 1,236 | -- | N/A | ^^ (I) | ^ | N/A | N/A | N/A | No systematic effect detected (p = 0.385); however, the analysis is based on only 2 specialty care interventions | N/A | N/A | Very low |
| **KQ1d. Patient outcomes** |  |  |  |  |  |  |  |  |  |  |  |  |
| **Provider intervention vs UCP** |  |  |  |  |  |  |  |  |  |  |  |  |
| Mean difference in depression rating scale scores | 9 RCTs [51, 52, 58, 61, 62, 79, 80, 83, 86]  N = 2,196 | -- | ^ (D) | D | (P) | N/A | N/A | N/A | Provider interventions not statistically significantly different from comparator groups (SMD -0.06; CI -0.14, 0.01) | N/A* | -- | Moderate |
| Odds of depression treatment response | 6 RCTs [52, 57, 60, 61, 64, 80]  N = 1,312 | -- | ^ (D) | D | (P) | n.s. | 189/591 | 252/721 | Provider interventions statistically significantly different from comparator groups (OR 1.12; CI 1.04, 1.21) favoring the intervention | 338/1000 | 24 more per 1000 | Moderate |
| Odds of depression recovery | 6 RCTs [52, 57, 60, 61, 79, 80]  N = 1,274 | -- | ^ (D) | D | (P) | N/A | 142/601 | 157/673 | Provider interventions not statistically significantly different from comparator groups (OR 1.02; CI 0.91, 1.15) | 248/1000 | n.s. | Moderate |
| Odds of depression treatment adherence | 2 RCTs [62, 83]  N = 281 | -- | -- | D | ^ | N/A | 47/130 | 70/151 | Provider interventions not statistically significantly different from comparator groups (OR 1.52; CI 0.70, 3.31) | 363/1000 | n.s. | Moderate |
| **Provider intervention vs system redesign** |  |  |  |  |  |  |  |  |  |  |  |  |
| Mean difference in depression rating scale scores | 3 RCTs [52, 53, 58]  N = 861 | -- | -- | D | ^ | N/A | N/A | N/A | Provider interventions not statistically significantly different from comparator groups (SMD 0.09; CI -0.48, 0.67) | N/A* | n.s. | Moderate |
| Odds of depression treatment response | 2 RCTs [52, 53]  N = 478 | -- | -- | D | ^ | N/A | N/A | N/A | Provider interventions not statistically significantly different from comparator groups (OR 0.53; CI 0.01, 40.38) | N/A | n.s. | Moderate |
| Odds of depression recovery | 2 RCTs [52, 53]  N = 478 | -- | -- | D | ^ | N/A | N/A | N/A | Provider interventions not statistically significantly different from comparator groups (OR 0.41; CI 0.01, 17.89) | N/A | n.s. | Moderate |
| Odds of depression treatment adherence | 1 RCT [53]  N = 61 | -- | ^^ (S) | D | (P) | N/A | N/A | N/A | Provider interventions not statistically significantly different from comparator groups (OR 0.16; CI 0.02, 1.39) | N/A | n.s. | Very low |
| **Provider intervention vs other interventions** |  |  |  |  |  |  |  |  |  |  |  |  |
| Odds of depression treatment adherence | 1 RCT [59]  N = 171 | -- | ^^ (S) | D | ^ | N/A | 53/85 | 48/86 | Provider intervention not statistically significantly different from motivational interviewing (OR 0.79; CI 0.30, 2.08) | 620/1000 | n.s. | Very low |
| Mean difference in treatment adherence | 1 RCT [59]  N = 171 | -- | ^^ (S) | D | (P) | N/A | 3.05 | 1.84 | Provider intervention not statistically significantly different from motivational interviewing (SMD -0.43; CI -0.76, -0.11) | N/A | -1.21 | Very low |

Notes: For GRADE, the following were consider: *study limitations* (low, medium, or high risk of bias), *indirectness* (direct or indirect), *inconsistency* (consistent, inconsistent, or unknown), *imprecision* (precise or imprecise), and *reporting bias* (likely present or not applicable). ^ downgrade by one, ^^ downgrade by two; D direct, PND power not discussed in study; IP insufficient power; (H) heterogeneity, (D) direction of effects, (S) single study, (I) indirect; (P) precise; N/A not applicable or not available; NC not able to be computed; IRR incidence rate ratio; OR odds ratio; SMD standardized mean difference; UCP usual care practice; vs versus; Poor RoB study rated with poor quality; * the outcome is a composite outcome and there is no meaningful absolute control risk score; n.s. not significant.
